# Glucuronolactone Promotes Mucin Sulfation to Alleviate Deoxynivalenol‐Induced Intestinal Injury via Microbiota‐Dependent and ‐Independent AHR Activation

**DOI:** 10.1002/advs.202522912

**Published:** 2026-02-23

**Authors:** Chenbin Cui, Beibei Zhang, Jiaxi Tang, Jing Hou, Yueqin Qiu, Kaiguo Gao, Li Wang, Zongyong Jiang, Xuefen Yang

**Affiliations:** ^1^ State Key Laboratory of Swine and Poultry Breeding Key Laboratory of Animal Nutrition and Feed Science in South China Ministry of Agriculture and Rural Affairs Guangdong Provincial Key Laboratory of Animal Breeding and Nutrition Institute of Animal Science Guangdong Academy of Agricultural Sciences Guangzhou China; ^2^ Heyuan Branch Guangdong Laboratory for Lingnan Modern Agriculture Heyuan China

**Keywords:** aryl hydrocarbon receptor, deoxynivalenol, glucuronolactone, intestinal microbiota, mucin sulfation

## Abstract

Deoxynivalenol (DON), a prevalent trichothecene mycotoxin, poses a global threat to the gut health of both humans and livestock. This study investigates the protective effects and underlying mechanisms of glucuronolactone (GLU) against DON‐induced intestinal injury. In a piglet model, GLU effectively alleviated DON‐induced intestinal injury and inflammation. Transcriptomic analysis revealed that GLU promotes mucin sulfation, a critical process for fortifying the intestinal mucus barrier. On the one hand, integrated microbiome and metabolomics analyses uncovered that GLU increased probiotic *Lactobacillus amylovorus* abundance and luminal indole‐3‐acetic acid level, thereby facilitating mucin sulfation. On the other hand, GLU itself directly boosted mucin sulfation in a microbiota‐independent manner. Mechanistically, both the microbiota‐dependent and ‐independent pathways through which GLU promoted mucin sulfation converged on the activation of aryl hydrocarbon receptor (AHR). Activated AHR transcriptionally up‐regulated the expression of the sulfotransferase *GAL3ST3*, which drove mucin sulfation. This study identifies GLU as a promising nutritional intervention against DON‐induced intestinal injury and reveals AHR‐mediated mucin sulfation as a vital mechanism for maintaining intestinal barrier homeostasis.

## Graphical abstract

Glucuronolactone (GLU) as a natural metabolite of glucose, increases *Lactobacillus amylovorus* abundance and luminal IAA level to activate AHR signaling. In addition, GLU itself can directly elevate AHR signaling activity independently of microbiota and IAA. AHR activation by GLU transcriptionally boosts sulfotransferase *GAL3ST3* expression to promote mucin sulfation, which strengthens the gut barrier integrity and eventually attenuates DON‐induced intestinal injury.

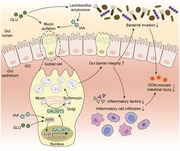

## 1 Introduction

Deoxynivalenol (DON), a prevalent trichothecene mycotoxin produced by *Fusarium* species, is a frequent contaminant in grain crops such as wheat and corn [1]. It has been reported that DON contamination rates were as high as 64% in grain samples worldwide and 33.3% to 100% in complete feed samples from China [2, 3]. Owing to its stability during food and feed processing, DON exposure is nearly unavoidable for both humans and livestock. The small intestine is the primary site for DON exposure and absorption. Both chronic and acute exposure can induce severe intestinal injury and immune dysfunction, thereby compromising intestinal health [4]. The intestinal toxicity of DON is mediated through multiple mechanisms, including oxidative stress induction and protein synthesis inhibition [5]. Consequently, developing effective strategies to mitigate DON‐induced intestinal injury is crucial for safeguarding public health and sustainable animal production.

Following absorption in the small intestine, DON can induce substantial alterations to key intestinal components such as epithelial cells (enterocytes, goblet cells, and stem cells) and the luminal microbiota [6]. As a specialized population of epithelial cells in the intestinal mucosa, goblet cells are an essential part of intestinal innate immunity [7]. Functional goblet cells play a pivotal role in maintaining gut homeostasis by secreting mucins, the key constituents of the protective mucus layer, thus regulating intestinal microbiota homeostasis and preventing the direct contact between luminal antigens and intestinal tissues [7]. Intestinal microbiota could be of great importance to DON intestinal toxicity since fecal microbiota transplantation from DON‐challenged mice to null mice causes severe intestinal injury and inflammation [8]. Notably, goblet cell dysfunction is closely associated with the progression of various gut diseases such as inflammatory bowel disease (IBD), colorectal cancer, and pathogen infections [9]. Goblet cell‐derived highly‐glycosylated mucins can be divided into neutral mucins and acidic mucins, and the acidic mucins comprise sulfomucin and sialomucin subtypes [10]. Among the multiple types of mucins, sialomucins are the most susceptible to bacterial enzymatic degradation, whereas sulfomucins are highly resistant [11]. Multiple studies have demonstrated that DON challenge triggers the loss of goblet cell number in the small intestine of both mice and piglets [12, 13]. Hence, goblet cell homeostasis may represent a promising therapeutic target for mitigating DON‐induced intestinal injury.

Glucuronolactone (GLU), a natural metabolite derived from glucose, has gained increasing attention due to its immunoregulatory properties [14]. GLU has been shown to effectively alleviate oxidative stress and cellular apoptosis induced by mycotoxin ochratoxin A [15]. In addition, our previous work has demonstrated that GLU can restore the intestinal barrier and redox balance to attenuate intestinal dysfunction induced by weaning stress [16]. However, whether GLU could ameliorate DON‐induced intestinal injury and the underlying mechanisms remain unknown.

In this study, we employed a multi‐model approach, including piglet and mouse in vivo models, porcine intestinal organoids, and a porcine intestinal epithelial cell line, to investigate the protective effects of GLU on DON‐induced intestinal injury. Additionally, we performed an integrated transcriptome‐microbiome‐metabolomics analysis to elucidate the underlying protective mechanisms of GLU. Our results demonstrate that GLU alleviates DON‐induced intestinal injury by effectively promoting mucin sulfation in the ileum. GLU‐associated microbiota and indole‐3‐acetic acid (IAA), as well as GLU itself, can activate aryl hydrocarbon receptor (AHR) signaling to transcriptionally facilitate sulfotransferase *GAL3ST3* expression, thereby promoting mucin sulfation. Collectively, these findings reveal a novel mechanism through which GLU improves intestinal homeostasis and underscore the therapeutic potential of GLU in mitigating DON‐induced intestinal injury.

## 2 Results

### 2.1 GLU Attenuates DON‐Induced Intestinal Injury and Inflammation

**FIGURE 1:**
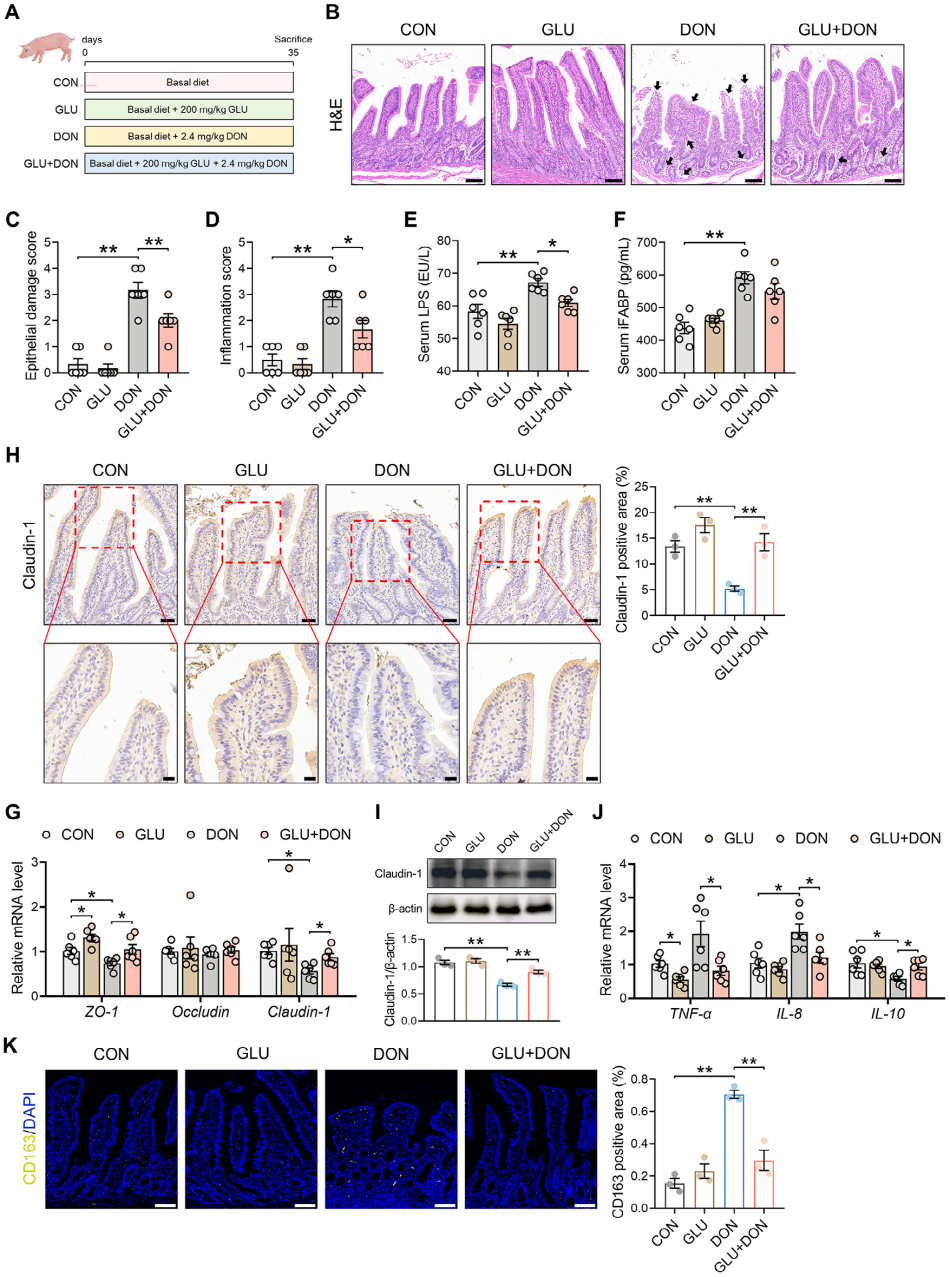
GLU alleviates DON‐induced intestinal injury and inflammation. (A) Piglet experimental scheme created with BioGDP.com. (B) H&E staining revealed the intestinal injury in the ileum of piglets, including submucosal edema, epithelial layer damage, and infiltration of inflammatory cells (black arrows). Scale bar: 100 µm. (C,D) The epithelial damage score and inflammation score in porcine ileum. *n* = 6. (E) LPS level in porcine serum. *n* = 6. (F) iFAPB level in porcine serum. *n* = 6. *n* = 4. (G) The mRNA expression of tight junction *ZO‐1*, *Occludin*, and *Claudin‐1* in porcine ileum. *n *= 6. (H) Representative images of Claudin‐1 staining and the measurement of the Claudin‐1 positive area. Scale bars: 100 µm (up) and 20 µm (down). *n* = 3. (I) Western blot assay of Claudin‐1 in porcine ileum. *n* = 3. (J) The mRNA expression of inflammatory factor *TNF‐α*, *IL‐8*, and *IL‐10* in porcine ileum. *n* = 6. (K) Representative images of CD163 staining (yellow) and the measurement of CD163 positive area. Scale bar: 100 µm. *n* = 3. Data are presented as mean ± SEM. Statistical analysis was performed using one‐way ANOVA with Tukey's multiple comparisons test. ^*^ *P* < 0.05 and ^**^ *P* < 0.01. H&E, hematoxylin and eosin; LPS, lipopolysaccharide; iFABP, intestinal fatty acid‐binding protein; TNF, tumor necrosis factor; IL, interleukin; DAPI, 4',6‐diamidino‐2‐phenylindole.

To investigate the therapeutic effect of GLU on DON‐induced intestinal injury, piglets received diets supplemented with 200 mg/kg GLU and/or 2.4 mg/kg DON (Figure 1A). Histological analysis of the ileum revealed that DON triggered severe pathological changes, including submucosal edema, epithelial layer damage, and infiltration of inflammatory cells in the lamina propria, while GLU supplementation effectively attenuated these pathological changes in the ileum (Figure 1B). Compared with piglets in the CON group, piglets in the DON group exhibited increased epithelial damage score and inflammation score, and additional GLU treatment significantly reduced epithelial damage score and inflammation score in the ileum (Figure 1C,D). We then examined the barrier function of the intestinal epithelium. DON piglets displayed elevated levels of serum lipopolysaccharide (LPS) and intestinal fatty acid‐binding protein (iFABP) compared with CON piglets, while GLU supplementation dramatically lowered serum LPS level (Figure 1E,F). Immunohistochemistry, western blot, and qPCR analyses revealed that DON down‐regulated the expression of tight junction protein ZO‐1 and Claudin‐1, while additional GLU treatment enhanced their expression levels in the ileum (Figure 1G–I). Besides, we assessed the inflammatory status in the ileum of piglets. We found that DON induced the expression of pro‐inflammatory factors interleukin (*IL*)*‐8* and inhibited the expression of anti‐inflammatory factor *IL‐10*, and additional GLU treatment reduced tumor necrosis factor (*TNF*)*‐α* and *IL‐8* expression and elevated *IL‐10* expression (Figure 1J). Immunofluorescence assay of a porcine macrophage marker CD163 demonstrated that compared with piglets in the CON group, piglets in the DON group exhibited increased area of CD163 positive cells in the ileal lamina propria, and GLU supplementation reduced the area of CD163 positive cells (Figure 1K), suggesting the decreased macrophage recruitment by GLU. Collectively, these results indicate that GLU can attenuate DON‐induced intestinal injury and inflammation.

### 2.2 GLU Promotes Mucin Sulfation to Alleviate DON‐Induced Intestinal Injury

**FIGURE 2:**
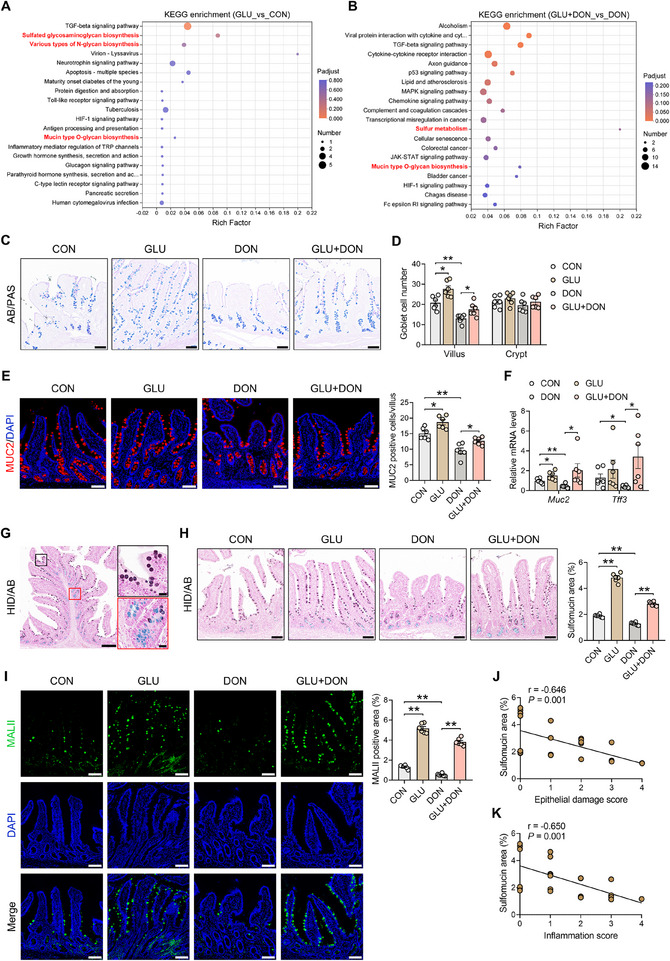
GLU boosts mucin sulfation to attenuate DON‐induced intestinal injury. (A,B) RNA sequencing was used to detect DEGs. KEGG enrichment analysis of DEGs in GLU versus CON and GLU+DON versus DON. *n* = 6. (C) Representative images of AB/PAS staining. Scale bar: 100 µm. (D) The quantification of goblet cells in the ileal villus and crypt. *n* = 6. (E) Representative images of MUC2 staining (red) and the quantification of MUC2‐positive cells (goblet cells) in the ileal villus. Scale bar: 100 µm. *n* = 6. (F) The mRNA expression of *MUC2* and *TFF3* in porcine ileum. *n *= 6. (G) HID/AB staining revealed the location of sulfomucin‐containing goblet cells and sialomucin‐containing goblet cells. Scale bars: 200 µm (left) and 100 µm (right). (H) Representative images of HID/AB staining and the quantification of sulfomucin area. Scale bar: 100 µm. *n* = 6. (I) Representative images of MALII staining (green) and the quantification of MALII‐positive area. Scale bar: 100 µm. *n* = 6. (J,K) Correlation analysis between the sulfomucin area and the epithelial damage score or the inflammation score. Data are presented as mean ± SEM. Statistical analysis was performed using one‐way ANOVA with Tukey's multiple comparisons test. ^*^ *P* < 0.05 and ^**^ *P* < 0.01. KEGG, Kyoto Encyclopedia of genes and genomes; DEG, differentially expressed gene; AB/PAS, alcian blue/periodic acid Schiff; MUC, mucin; TFF, trefoil factor family; HID/AB, iron diamine/alcian blue; DAPI, 4',6‐diamidino‐2‐phenylindole.

We next performed RNA sequencing on porcine intestinal epithelium to explore the underlying mechanism through which GLU improves DON‐induced intestinal injury and inflammation. Principal components analysis (PCA) was used to describe the characteristic of transcriptome data among groups, and the cluster analysis and volcano plot were used to visualize the distribution of differential expressed genes (DEGs) among groups. The results showed that GLU supplementation triggered substantial alterations in gene expression under both homeostasis conditions and DON challenge (Figure S1A–C). Kyoto encyclopedia of genes and genomes (KEGG) pathway analysis revealed that DEGs in both GLU versus CON group and GLU+DON versus DON group were primarily enriched in pathways associated with goblet cell‐derived mucins, such as various types of N‐glycan biosynthesis and mucin type O‐glycan biosynthesis (Figure 2A,B). Therefore, we subsequently examined goblet cell homeostasis in the ileum. Alcian blue/periodic acid Schiff (AB/PAS) staining revealed that DON challenge lowered goblet cell number in the villi of the ileum, while GLU treatment increased goblet cell number in ileal villi under both homeostasis conditions and DON challenge (Figure 2C,D). GLU and/or DON treatment had no effect on goblet cell number in the crypts of the ileum (Figure 2D). Immunofluorescence and qPCR analyses of goblet cell markers mucin (MUC) 2 and trefoil factor family (TFF) 3 were conducted to further validate the finding. We found that MUC2‐positive cell number in villus, as well as *MUC2* and *TFF3* expression, was also significantly elevated by GLU treatment (Figure 2E,F).

In the KEGG pathway analysis of intestinal epithelium transcriptome, we noticed that GLU‐induced DEGs were also enriched in pathways associated with sulfur, such as sulfated glycosaminoglycan biosynthesis and sulfur metabolism (Figure 2A,B), which reminded us of the goblet cell‐derived sulfomucins. Using high iron diamine/alcian blue (HID/AB) staining, we found that sulfomucin‐containing goblet cells were mainly scattered at the villi, and sialomucin‐containing goblet cells resided in the crypts in the ileum (Figure 2G). The sulfomucin abundance was reduced in the ileal epithelium in DON piglets compared with CON piglets, while GLU supplementation markedly increased the sulfomucin abundance in the ileal epithelium (Figure 2H). In addition, the staining of *Maackia Amurensis* lectin II (MALII) recognizing α2,3‐linked sialylated and sulfated glycans allows for the visualization of sulfated O‐glycans on MUC2 [17]. We found that MALII abundance was also elevated by GLU treatment under homeostasis and DON conditions (Figure 2I). Notably, our analysis revealed that sulfomucin abundance negatively correlated with epithelial damage score and inflammation score in the ileum (Figure 2J,K). Taken together, these findings suggest that GLU can promote mucin sulfation to alleviate DON‐induced intestinal injury.

To further confirm the effect of GLU on mucin sulfation and determine the appropriate dosage of GLU on mice, we treated mice with 40 mg/kg (LGLU group) or 80 mg/kg (HGLU group) GLU via gavage (Figure S2A). Both 40 and 80 mg/kg GLU had no effect on ileal morphology (Figure S2B). However, the mice in the HGLU group exhibited increased mRNA expression of *TNF‐α* (Figure S2C). Although GLU treatment did not alter goblet cell number in murine ileum (Figure S2D,E), the sulfomucin abundance was dramatically elevated in LGLU and HGLU groups compared with the CON group (Figure S2F). Therefore, we treat mice with 40 mg/kg GLU in the subsequent experiments.

### 2.3 GLU‐Associated Gut Microbiota Supports Mucin Sulfation and Alleviates DON‐Induced Intestinal Injury

**FIGURE 3:**
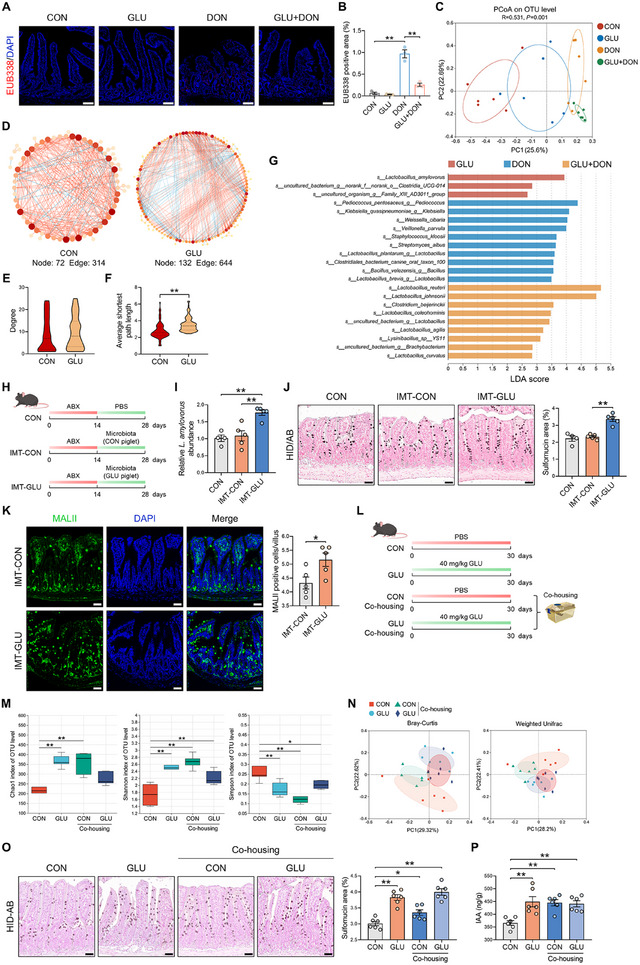
GLU alters the gut microbiota to facilitate mucin sulfation. (A,B) Representative images of FISH assay (EUB338, red) in porcine ileum and the quantification of EUB338‐positive area. Scale bar: 100 µm. *n* = 3. (C) The PCoA plot of 16s rRNA sequencing data from porcine ileal contents based on Bray–Curtis analysis. *n* = 6. (D) Network analysis of the ileal microbiota in piglets. (E,F) Comparison of network degree and average shortest path length. (G) LEfSe analysis with an LDA score > 2.5 of porcine ileal microbiota. *n *= 6. (H) IMT experimental scheme created with BioGDP.com. (I) *Lactobacillus amylovorus* abundance in murine feces. *n* = 4–5. (J) Representative images of HID/AB staining and the quantification of sulfomucin area in the IMT experiment. Scale bar: 50 µm. *n* = 4–5. (K) Representative images of MALII staining (green) and the quantification of MALII‐positive cells per villus in the IMT experiment. Scale bar: 50 µm. *n* = 5. (L) Co‐housing experimental scheme created with BioGDP.com. (M) α‐diversity (assessed by Chao1 index, Shannon index, and Simpson index) of the ileal microbiota in the co‐housing experiment. *n* = 6. (N) The PCoA plot of 16s rRNA sequencing data from murine ileal contents based on Bray–Curtis and weighted Unifrac analysis in the co‐housing experiment. *n* = 6. (O) Representative images of HID/AB staining and the quantification of sulfomucin area in the co‐housing experiment. Scale bar: 50 µm. *n* = 6. (P) IAA level in the murine level in the co‐housing experiment. *n* = 6. Data are presented as mean ± SEM. Statistical analysis was performed using one‐way ANOVA with Tukey's multiple comparisons test, except for E and F. ^*^ *P* < 0.05 and ^**^ *P *< 0.01. FISH, fluorescence in situ hybridization; PCoA, Principal coordinate analysis; IMT, ileal microbiota transplant; HID/AB, iron diamine/alcian blue; DAPI, 4',6‐diamidino‐2‐phenylindole. IAA, indole‐3‐acetic acid.

Considering that goblet cells can modulate intestinal microbiota via mucin secretion [7], we therefore investigated the homeostasis of ileal microbiota in piglets following treatment with GLU and/or DON. Fluorescence in situ hybridization (FISH) assay with a universal bacterial probe revealed a marked increase in bacterial colonization in the ileum of DON‐challenged piglets, and additional GLU treatment reduced bacterial colonization (Figure 3A,B). Besides, we performed 16S rRNA sequencing to examine the composition and structure of ileal microbiota in piglets. Generally, α‐diversity (such as Chao1, Shannon, and Simpson indices) is used to examine the within‐sample richness and diversity of microbiota, and β‐diversity (such as principal coordinate analysis (PCoA) analysis) aims to analyzing the between‐sample differences of species composition. PCoA demonstrated significant distinctions in bacterial profiles between the four groups (Figure 3C), while the Chao1, Shannon, and Simpson indices among these groups exhibited no significant differences (Figure S3A), suggesting that GLU and/or DON changes species composition rather than overall microbial richness and diversity. GLU and/or DON treatment altered the microbial composition on both the phylum level and genus level (Figure S3B,C). Microbial co‐occurrence network analysis is a visual and structural tool to illustrate the compositional complexity and potential interaction patterns within the microbiota [18]. We found that the GLU group had more nodes and edges than the CON group (Figure 3D). At the node level, the average shortest path length of the GLU group was markedly higher than that of the CON group (Figure 3E), and the degree displayed no significant difference between the CON and GLU groups (Figure 3F). Linear discriminant analysis effect size (LEfSe) analysis revealed that probiotic *Lactobacillus* such as *Lactobacillus amylovorus* (*L. amylovorus*) and *Lactobacillus reuteri* (*L. reuteri*) were enriched in the GLU‐treated groups, and pathogenic *Klebsiella quasipneumoniae* was abundant in the DON group (Figure 3G). These findings suggest that GLU and/or DON treatment results in marked alterations in ileal microbial composition in piglets.

The gut microbiota plays an important role in multiple biological processes [19, 20]. As the microbiota of GLU piglets was altered in comparison with CON piglets, we investigated whether GLU‐associated microbiota contributed to the strengthened mucin sulfation. We colonized antibiotic (ABX)‐pretreated mice with ileal microbiota from either CON or GLU piglets (Figure 3H) and analyzed the effects of ileal microbiota transplant (IMT) on mucin sulfation. IMT from GLU‐treated piglets increased *L. amylovorus* abundance in the feces of recipient mice (Figure 3I). HID‐AB staining demonstrated that the sulfomucin area in the IMT‐GLU group was larger than that in the IMT‐CON group (Figure 3J). In addition, MALII staining also revealed the increased sulfomucin abundance in the IMT‐GLU group compared with the IMT‐CON group (Figure 3K). We next performed a co‐housing experiment to further confirm the beneficial effect of GLU‐associated microbiota on mucin sulfation (Figure 3L). Compared with separately‐housed CON mice, separately‐housed GLU mice exhibited increased Chao1 index and Shannon index and decreased Simpson index in the ileal microbiota (Figure 3M). Co‐housing CON mice with GLU mice enhanced the Chao1 index and Shannon index and lowered the Simpson index in CON mice (Figure 3M). What's more, PCoA analysis revealed the different β‐diversity between separately‐housed CON mice and separately‐housed GLU mice and the similar β‐diversity between co‐housed CON mice and co‐housed GLU mice (Figure 3N). These data indicate that co‐housing treatment offers CON mice a similar microbiota to GLU mice. Notably, we found that compared with separately‐housed CON mice, co‐housed CON mice displayed elevated sulfomucin abundance in the ileum (Figure 3O). Collectively, these results suggest that the altered gut microbiota induced by GLU contributes to mucin sulfation.

To evaluate the effect of GLU‐associated microbiota on DON‐induced intestinal injury, DON was given to null mice with or without co‐housing with GLU mice (Figure S4A). We found that after co‐housing with GLU mice, null mice exhibited improved intestinal injury (Figure S4B) and decreased epithelial damage score and inflammation score (Figure S4C,D). Besides, co‐housing treatment with GLU mice also lowered the level of serum LPS in DON‐challenged null mice (Figure S4E). These findings demonstrate that GLU‐associated microbiota can alleviate DON‐induced intestinal injury.

### 2.4 Lactobacillus Amylovorus Facilitates IAA Production to Support Mucin Sulfation

**FIGURE 4:**
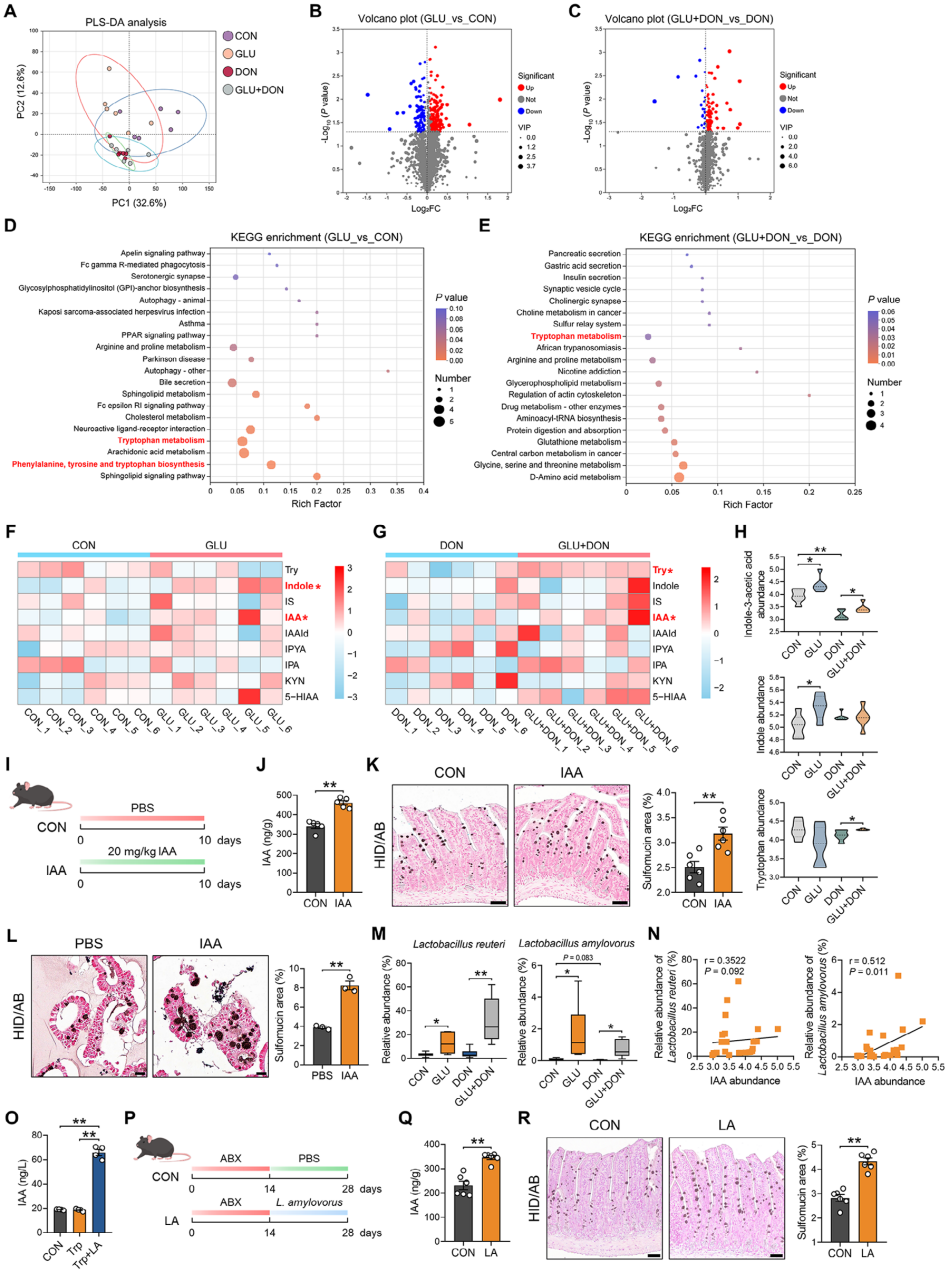
*Lactobacillus amylovorus* facilitates IAA production to support mucin sulfation. (A) The PLS‐DA plot of metabolomics data from porcine ileal contents. *n* = 6. (B,C) The volcano plot (fold change > 1, *P* < 0.05) for differential metabolites in GLU versus CON and GLU+DON versus DON. Red dots: significantly up‐regulated genes, blue dots: significantly down‐regulated genes. *n* = 6. (D,E) KEGG enrichment analysis of differential metabolites in GLU versus CON and GLU+DON versus DON. *n* = 6. (F,G) Heatmap of metabolites associated with tryptophan metabolism in GLU versus CON and GLU+DON versus DON. *n* = 6. (H) The abundances of IAA, indole, and tryptophan in the porcine ileum. *n* = 6. (I) IAA experimental scheme created with BioGDP.com. (J) IAA levels in murine ileal contents. *n* = 5. (K) Representative images of HID/AB staining and the quantification of sulfomucin area in the IAA experiment. Scale bar: 50 µm. *n* = 6. (L) Representative images of HID/AB staining and the quantification of sulfomucin area in PBS or IAA‐treated organoids. Scale bar: 20 µm. *n* = 3. (M) The relative abundances of *Lactobacillus reuteri* and *Lactobacillus amylovorus* in the porcine ileum. *n *= 6. (N) Correlation analysis between IAA and *Lactobacillus reuteri* or *Lactobacillus amylovorus*. (O) IAA levels in the media. *n* = 4. (P) *Lactobacillus amylovorus* experimental scheme created with BioGDP.com. (Q) The IAA level in the murine ileum in the *Lactobacillus amylovorus* experiment. *n* = 6. (R) Representative images of HID/AB staining and the quantification of sulfomucin area in the *Lactobacillus amylovorus* experiment. Scale bar: 50 µm. *n* = 6. Data are presented as mean ± SEM. Statistical analysis was performed using unpaired Student's *t*‐test and one‐way ANOVA with Tukey's multiple comparisons test. ^*^ *P* < 0.05 and ^**^ *P* < 0.01. IAA, indole‐3‐acetic acid; PLS‐DA, partial least squares discriminant analysis; HID/AB, iron diamine/alcian blue; LA, *Lactobacillus amylovorus*.

It has been generally accepted that the gut microbiota can manipulate luminal metabolites to modulate intestinal homeostasis [21, 22]. To explore the potential metabolite affecting mucin sulfation in GLU‐associated microbiota, metabolomics analysis was performed on the ileal contents of piglets. We found that GLU and/or DON treatment resulted in significant alterations in the ileal metabolite profile (Figure 4A). Compared with the CON group, 200 metabolites were up‐regulated and 82 metabolites were down‐regulated in the GLU group (Figure 4B). Compared with the DON group, 104 metabolites were up‐regulated, and 22 metabolites were down‐regulated in the GLU+DON group (Figure 4C). Notably, KEGG pathway analysis revealed that the differential metabolites in the GLU versus CON group and GLU+DON versus DON group were enriched in pathways associated with tryptophan, such as tryptophan metabolism and phenylalanine, tyrosine, and tryptophan biosynthesis (Figure 4D,E). Therefore, we then analyzed the abundances of tryptophan metabolites in different groups. Among various tryptophan metabolites, GLU supplementation contributed to significant increases in indole and IAA abundances under homeostasis (Figure 4F) and in tryptophan and IAA abundances under DON challenge (Figure 4G). DON challenge reduced the abundance of IAA rather than indole and tryptophan (Figure 4H). In the co‐housing experiment, GLU treatment up‐regulated IAA level in the ileal contents, and co‐housing CON mice with GLU mice also led to increased IAA level in CON mice (Figure 3P), further suggesting the potential relation between GLU‐associated microbiota and IAA. We next validated the effect of IAA on mucin sulfation in mice (Figure 4I). IAA (20 mg/kg) supplementation for 10 days elevated luminal IAA level which was similar to that in GLU‐treated mice in Figure 3P, reflecting physiologically relevant concentration produced by GLU‐associated microbiota (Figure 4J). As expected, IAA dramatically boosted sulfomucin abundance in the ileum (Figure 4K). In addition, we isolated porcine intestinal crypts and established porcine intestinal organoids for IAA treatment. The sulfomucin abundance was also elevated by IAA treatment in intestinal organoids (Figure 4L). These data indicate that IAA mediates the mucin sulfation‐promoting effect of GLU.

Subsequently, we aimed to identify the bacteria facilitating IAA production in GLU‐treated piglets. It has been reported that *Lactobacillus*, such as *L. reuteri* and *L. amylovorus*, contributes to IAA production in the gut [23, 24]. In consideration of the enrichment of *L. reuteri* and *L. amylovorus* in GLU‐treated groups (Figure 3G), we analyzed the two bacteria and found that DON challenge led to a decreased trend of *L. amylovorus*, and GLU could increase the abundances of *L. reuteri* and *L. amylovorus* under both homeostasis and DON conditions (Figure 4M). Notably, our analysis revealed a significant positive correlation between IAA and *L. amylovorus* rather than *L. reuteri* (Figure 4N).

We then tested the effect of *L. amylovorus* on IAA production in vitro. *L. amylovorus* significantly increased IAA level in the media with tryptophan supplementation (Figure 4O). In addition, we orally administered *L. amylovorus* or PBS to ABX‐pretreated mice for 14 days (Figure 4P). Microbiota sequencing analysis revealed that *L. amylovorus* treatment had no effect on α‐diversity (Figure S5A) but altered β‐diversity and microbiota composition (Figure S5B,C) in the ileum. The IAA level was enhanced in the ileum after *L. amylovorus* administration (Figure 4Q), and the sulfomucin abundance was also elevated by *L. amylovorus* (Figure 4R). Taken together, our findings suggest that *L. amylovorus* can facilitate IAA production to support mucin sulfation.

To validate the protective effect of *L. amylovorus* against DON‐mediated intestinal toxicity, we evaluated mouse phenotypes after DON and *L. amylovorus* treatment (Figure S6A). Histological analysis of the ileum demonstrated that *L. amylovorus* administration effectively alleviated DON‐induced intestinal injury (Figure S6B) and lowered epithelial damage score and inflammation score (Figure S6C,D). Compared with mice in the DON group, mice in the LA+DON group displayed an increase in sulfomucin abundance (Figure S6E). Besides, macrophage marker F4/80 staining showed that the DON‐induced increase in F4/80‐positive area was reduced by *L. amylovorus* treatment (Figure S6F). These data indicate that *L. amylovorus* effectively attenuates DON‐mediated intestinal toxicity.

### 2.5 GLU Fosters Mucin Sulfation and Alleviates DON‐Induced Intestinal Injury in a Microbiota‐Independent Manner

**FIGURE 5:**
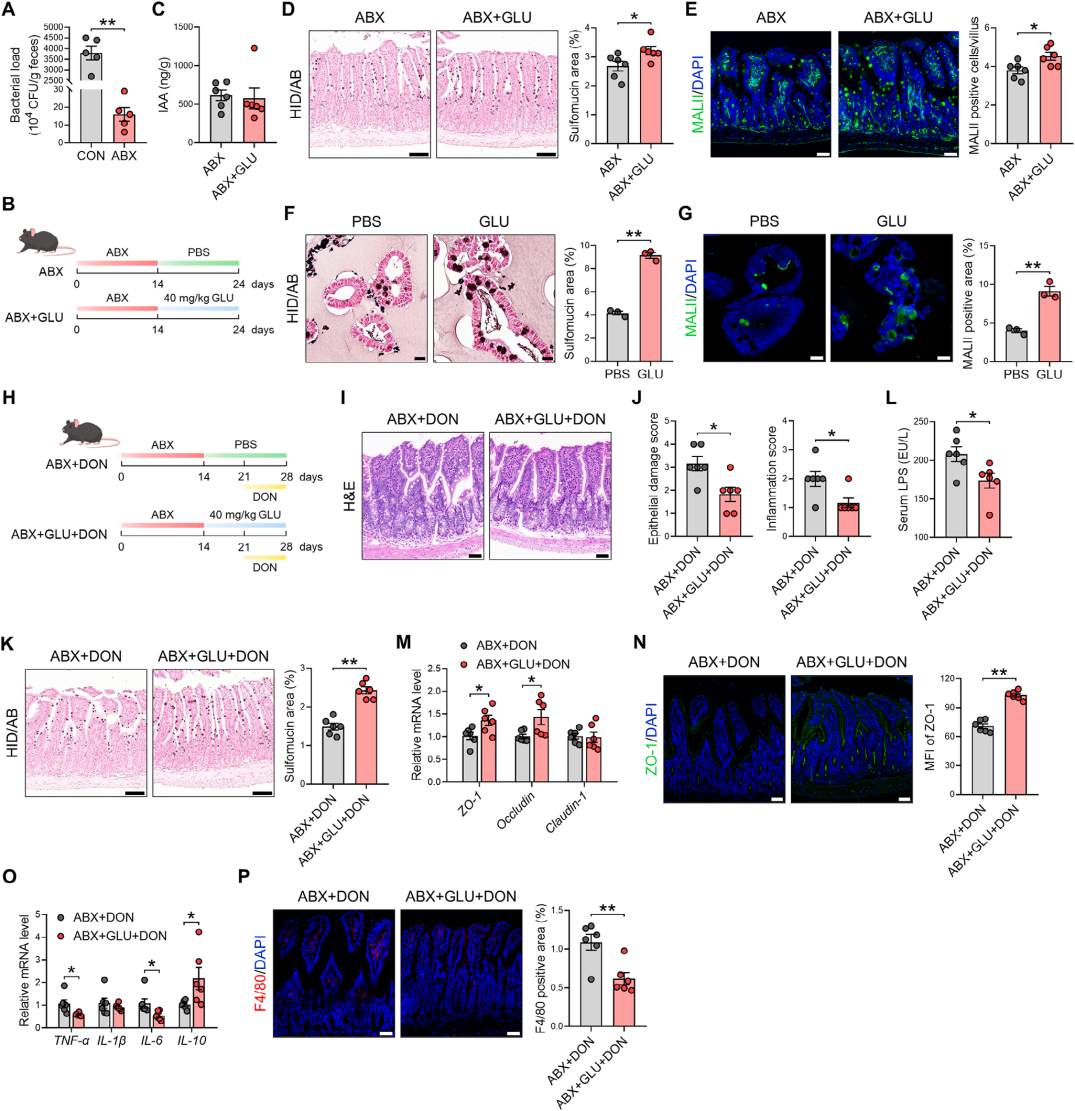
GLU fosters mucin sulfation and alleviates DON‐induced intestinal injury in a microbiota‐independent manner. (A) Fecal bacterial load in mice with or without ABX treatment. *n* = 5. (B) ABX and GLU experimental scheme created with BioGDP.com. (C) The IAA level in the murine ileum in the ABX and GLU experiment. *n* = 6. (D) Representative images of HID/AB staining and the quantification of sulfomucin area in the ABX and GLU experiment. Scale bar: 100 µm. *n* = 6. (E) Representative images of MALII staining (green) and the quantification of MALII‐positive cells per villus in murine ileum. Scale bar: 50 µm. *n* = 6. (F) Representative images of HID/AB staining and the quantification of sulfomucin area in PBS or GLU‐treated organoids. Scale bar: 20 µm. *n* = 3. (G) Representative images of MALII staining (green) and the quantification of MALII‐positive area in PBS or GLU‐treated organoids. Scale bar: 20 µm. *n* = 3. (H) ABX, GLU, and DON experimental scheme created with BioGDP.com. (I) Representative images of H&E staining in the murine ileum. Scale bar: 50 µm. (J) The epithelial damage score and inflammation score in the murine ileum. *n* = 6. (K) Representative images of HID/AB staining and the quantification of sulfomucin area in the murine ileum. Scale bar: 100 µm. *n* = 6. (L) The serum LPS level in mice. *n* = 6. (M) The mRNA levels of *ZO‐1*, *Occludin*, and *Claudin‐1* in the murine ileum. *n *= 6. (N) Representative images of ZO‐1 staining (green) and the quantification of ZO‐1 MFI in the murine ileum. Scale bar: 50 µm. *n* = 6. (O) The mRNA levels of *TNF‐α*, *IL‐1β*, *IL‐6*, and *IL‐10* in the murine ileum. *n* = 6. (P) Representative images of F4/80 staining (red) and the quantification of F4/80‐positive area in the murine ileum. Scale bar: 50 µm. *n* = 6. Data are presented as mean ± SEM. Statistical analysis was performed using an unpaired Student's *t*‐test. ^*^ *P* < 0.05 and ^**^ *P* < 0.01. ABX, antibiotic; HID/AB, iron diamine/alcian blue; H&E, hematoxylin and eosin; TNF, tumor necrosis factor; IL, interleukin; DAPI, 4',6‐diamidino‐2‐phenylindole.

Due to the immunoregulatory property of GLU [14], we speculated that GLU itself might improve mucin sulfation and DON‐induced intestinal injury. To investigated whether the distinct microbiota induced by GLU was essential for its mucin sulfation‐promoting effect, ABX treatment was used to remove intestinal microbiota (Figure 5A). ABX‐pretreated mice were gavaged with GLU for 10 days (Figure 5B). Interestingly, after microbiota elimination, although luminal IAA level was not altered by GLU (Figure 5C), GLU administration still up‐regulated sulfomucin abundance in the ileum as demonstrated by HID/AB staining and MALII staining (Figure 5D,E). To further confirm that GLU could promote mucin sulfation independently of gut microbiota, we performed GLT treatment on porcine intestinal organoids which were sterile. As expected, the sulfomucin abundance was also boosted by GLU administration in organoids (Figure 5F,G). We next wondered whether GLU could protect mice from DON‐induced intestinal injury without gut microbiota (Figure 5H). Histological analysis of the ileum revealed that GLU alleviated DON‐induced intestinal injury (Figure 5I) and decreased epithelial damage score and inflammation score (Figure 5J) in ABX‐pretreated mice. Sulfomucin abundance was elevated by GLU in ABX‐pretreated mice (Figure 5K). GLU treatment also restored intestinal barrier function as demonstrated by decreased serum LPS level (Figure 5L) and increased ZO‐1 and Occludin expression (Figure 5M,N). Additionally, intestinal inflammation status was improved by GLU as shown by the decreased pro‐inflammatory factor expression (Figure 5O) and F4/80 positive area (Figure 5P). Collectively, these findings suggest that GLU can foster mucin sulfation and attenuate DON‐induced intestinal injury independently of gut microbiota.

### 2.6 GLU Boosts Mucin Sulfation via AHR Activation

**FIGURE 6:**
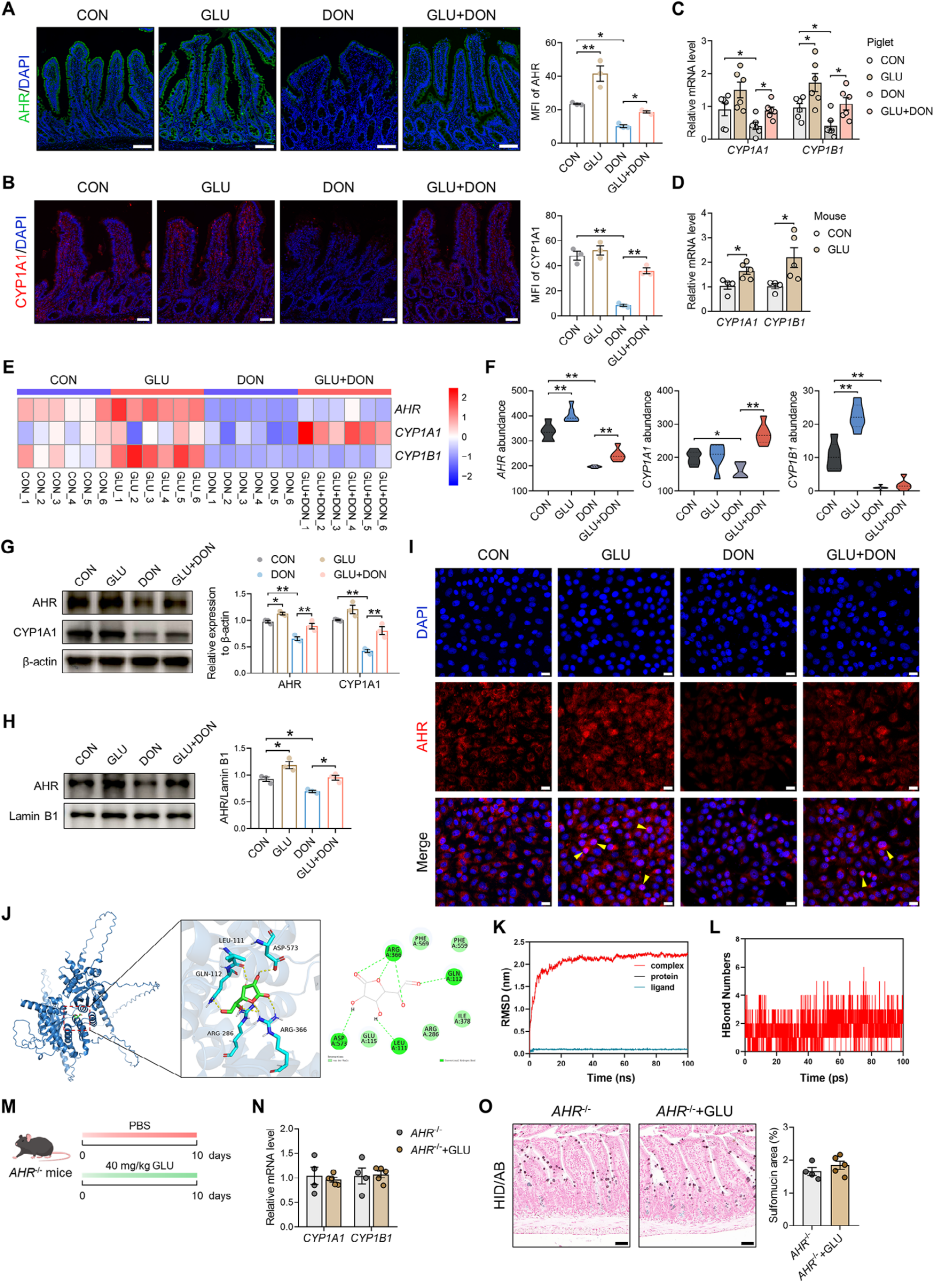
GLU boosts mucin sulfation via AHR activation. (A) Representative images of AHR staining (green) and the quantification of AHR MFI in the porcine ileum. Scale bar: 100 µm. *n* = 3. (B) Representative images of CYP1A1 staining (red) and the quantification of AHR MFI in the porcine ileum. Scale bar: 100 µm. *n* = 3. (C) The mRNA levels of *CYP1A1* and *CYP1B1* in the porcine ileum. *n* = 6. (D) The mRNA levels of *CYP1A1* and *CYP1B1* in the ileum of the murine GLU experiment. *n* = 4–5. (E,F) The expression of *AHR* and its target genes in the porcine intestinal epithelium. *n* = 6. (G) The protein levels of AHR and CYP1A1 in GLU and/or DON‐treated porcine intestinal epithelium were examined by immunoblot analysis. *n* = 3. (H) The nuclear protein in the porcine intestinal epithelium was isolated, and the nuclear AHR level was detected by immunoblot analysis. *n* = 3. (I) Representative images of AHR staining (red) in the porcine intestinal epithelium treated with GLU and/or DON. Scale bar: 20 µm. Yellow arrowheads represented nuclear AHR. (J) Molecular docking simulation for the ligand–protein binding between DON and porcine AHR. (K) RMSD analysis of the complex, protein, and ligand. (L) The number of hydrogen bonds in the ligand–protein complex. (M) AHR knockout and GLU experimental scheme created with BioGDP.com. (N) The mRNA levels of *CYP1A1* and *CYP1B1* in the ileum of AHR^−/−^ mice with or without GLU administration. *n* = 4–5. (O) Representative images of HID/AB staining and the quantification of sulfomucin area in the ileum of AHR^−/−^ mice with or without GLU administration. Scale bar: 50 µm. *n* = 4–5. Data are presented as mean ± SEM. Statistical analysis was performed using unpaired Student's *t‐*test and one‐way ANOVA with Tukey's multiple comparisons test. ^*^ *P* < 0.05 and ^**^ *P* < 0.01. DAPI, 4',6‐diamidino‐2‐phenylindole; RMSD, root mean square deviation; HBond, hydrogen bond; HID/AB, iron diamine/alcian blue.

We subsequently investigated the mechanism underlying the mucin sulfation‐promoting effects of GLU‐associated gut microbiota and GLU itself. It has been reported that AHR signaling plays an important role in mucin sulfation [25]. Immunostaining and qPCR analyses revealed that DON challenge weakened the expression of AHR and its target genes CYP1A1 and CYP1B1, and additional GLU supplementation restored their expression (Figure 6A–C). In mice, the expression of *CYP1A1* and *CYP1B1* was also up‐regulated by GLU administration (Figure 6D). Considering that IAA acts as an AHR ligand to activate AHR signaling, we assessed AHR signaling activity in murine LA and IAA supplementation experiments. GLU‐associated LA and IAA could enhance AHR signaling activity as demonstrated by the increased *CYP1A1* expression in murine ileum (Figure S7A–C). Then, we re‐analyzed the transcriptome data of porcine intestinal epithelium treated with DON and/or GLU. We found that DON lowered the gene expression of *AHR*, *CYP1A1*, and *CYP1B1*, while additional GLU treatment rescued the gene expression of *AHR* and *CYP1A1* (Figure 6E,F). Western blot assay also demonstrated that GLU restored the activity of AHR signaling after DON challenge (Figure 6G). What's more, we noticed that DON reduced nuclear AHR level, and additional GLU treatment increased nuclear AHR level, suggesting that GLU promotes the nuclear translocation of AHR (Figure 6H,I, yellow arrowheads). To elucidate the interaction between GLU and porcine AHR protein, we performed a molecular docking analysis. Docking results showed that GLU could bind to porcine AHR through visible hydrogen bonds and strong electrostatic interactions, with a low binding energy of −5.347 kcal/mol (Figure 6J). The hydrogen bonding between GLU and the residue LEU‐111, GLN‐112, ARG‐366, and ASP‐573 could play a crucial role in GLU‐AHR interaction (Figure 6J). Subsequent molecular dynamics simulations analysis on the GLU‐AHR complex also verified the binding capability of GLU to AHR with high stability (Figure 6K,L). Sequence homology verification of porcine AHR protein showed that all residues binding with GLU were highly conservative among various species (Figure S8A). In GLU‐AHR binding, AGR‐366 could be a key residue since GLU bound to it through three hydrogen bondings (Figure 6J). We then performed a drug affinity responsive target stability (DARTS) assay to investigate the binding between GLU and AHR AGR‐366. The result showed that the binding between GLU and AHR^WT^ increased the stability of AHR protein, while the AHR AGR‐366 mutation abolished the binding between GLU and AHR and the increased AHR stability (Figure S8B). In addition, we analyzed the binding between the known AHR ligand IAA and porcine AHR using molecular docking. The binding energy between IAA and porcine AHR was −5.5 kcal/mol, which was similar to that between GLU and porcine AHR. The hydrogen bonding between IAA and the residues TYR‐330, VAL‐348, and ARG‐396 could play a crucial role in IAA‐AHR interaction (Figure S9). Taken together, these findings indicate that GLU‐associated LA and IAA, as well as GLU itself, can activate the activity of AHR signaling.

Then, we utilized *AHR* knockout mice (*AHR^−/−^* mice) to explore the necessity of AHR in GLU‐mediated mucin sulfation (Figure 6M). GLU administration failed to increase the mRNA expression of *CYP1A1* and *CYP1B1* in *AHR^−/−^* mice (Figure 6N). Notably, the sulfomucin abundance was unchanged in GLU‐treated *AHR^−/−^* mice (Figure 6O). We also treated WT mice with AHR antagonist CH‐223191 to repress AHR activity (Figure S10A). The mRNA expression of *CYP1A1* and *CYP1B1* was not altered by GLU in CH‐223191‐treated mice (Figure S10B). AHR inhibition by CH‐223191 also abolished the mucin sulfation‐promoting effect of GLU (Figure S10C). Next, we wondered whether AHR inhibition could abrogate the beneficial effect of GLU on DON‐induced intestinal injury (Figure S10D). The results revealed that under the condition of AHR inhibition, GLU had no influence on DON‐induced intestinal injury (Figure S10E–G), as well as inflammation (Figure S10H,I). Taken together, these data suggest that GLU activates AHR signaling to boost mucin sulfation, thus alleviating DON‐induced intestinal disorders.

In addition, to figure out whether IAA‐mediated AHR signaling was involved in GLU‐mediated mucin sulfation, we examined the promoting effect of GLU on mucin sulfation in mice with or without ABX treatment which could remove microbiota to block IAA production (Figure S11A). The results showed that mice in the ABX+GLU group exhibited reduced sulfomucin abundance compared with mice in the GLU group (Figure S11B), suggesting that IAA signaling is involved in GLU‐mediated mucin sulfation. We then investigated whether IAA‐mediated AHR signaling was involved in the effects of GLU‐associated microbiota on mucin sulfation and DON‐induced intestinal injury. We additionally used AHR inhibitor CH‐223191 to inhibit mucin sulfation on the basis of the experimental treatments in Figure S4 (Figure S11C). We found that after co‐housing with GLU‐treated mice, null mice exhibited alleviated intestinal injury and reduced epithelial damage score and inflammation score, while additional CH‐223 191 treatment abolished the improvements in these indices (Figure S10D–F). Besides, co‐housing with GLU‐treated mice elevated sulfomucin abundance in DON‐challenged null mice, while additional CH‐223191 treatment eliminated the increase in sulfomucin abundance (Figure S11G). These findings demonstrate that IAA signaling is involved in the promoting effects of GLU or GLU‐associated microbiota on mucin sulfation.

### 2.7 GLU Enhances Sulfotransferase GAL3ST3 Expression via AHR‐Mediated Transcriptional Regulation

**FIGURE 7:**
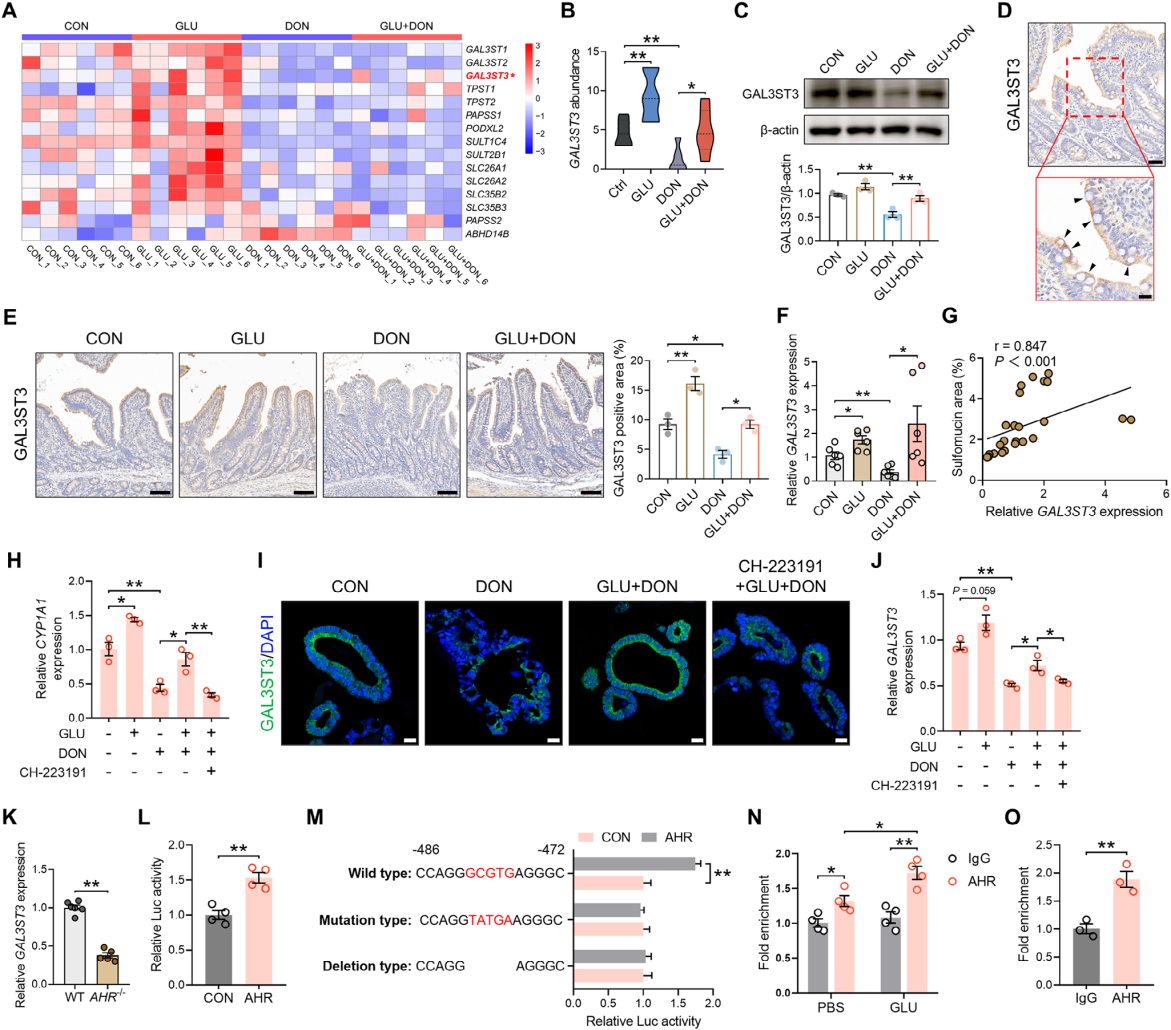
GLU enhances sulfotransferase GAL3ST3 expression via AHR‐mediated transcriptional regulation. (A) Heatmap of gene expression in the cytosolic sulfation pathway in the porcine intestinal epithelium. *n* = 6. (B) The abundance of *GAL3ST3* in the porcine intestinal epithelium. *n* = 6. (C) The protein level of GAL3ST3 in IPEC‐J2 cells treated with GLU and/or DON. *n* = 3. (D) GAL3ST3 staining revealed the robust GAL3ST3 expression in porcine goblet cells (black arrowheads). Scale bars: 50 µm (left) and 20 µm (right). (E) Representative images of GAL3ST3 staining and the quantification of GAL3ST3‐positive area in the porcine ileum. Scale bar: 100 µm. *n* = 3. (F) The *GLA3ST3* mRNA expression in the porcine ileum was examined by qPCR. *n* = 6. IPEC‐J2 cells or porcine intestinal organoids were treated with GLU, DON, and CH‐223191 (AHR antagonist) for 24 h. (G) Correlation analysis between sulfomucin area and relative *GAL3ST3* expression in porcine ileum. (H) The *CYP1A1* mRNA expression in IPEC‐J2 cells was examined by qPCR. *n* = 3. (I) Representative images of GAL3ST3 staining in porcine intestinal organoids. Scale bar: 20 µm. (J) The *GAL3ST3* mRNA expression in IPEC‐J2 cells was detected by qPCR. *n* = 3. (K) GAL3ST3 mRNA expression in WT and *AHR^−/−^* mice. *n* = 5–6. (L) Porcine *GAL3ST3* promoter reporter and pTK were co‐transfected with pCMV‐myc or pCMV‐AHR into HEK293T cells. After 24 h, the dual‐luciferase activity was detected. *n* = 4. (M) Porcine *GAL3ST3* wild type, mutated, and deleted promoter reporter and pTK were co‐transfected with pCMV‐myc or pCMV‐AHR into HEK293T cells. After 24 h, the dual‐luciferase activity was examined. *n* = 4. (N,O) CUT&RUN‐qPCR analysis was used to detect porcine *GAL3ST3* promoter from the DNA precipitated by anti‐AHR antibody in IPEC‐J2 cells or porcine small intestinal epithelial cells. *n* = 3–4. Data are presented as mean ± SEM. Statistical analysis was performed using unpaired Student's *t‐*test and one‐way ANOVA with Tukey's multiple comparisons test. ^*^ *P* < 0.05 and ^**^ *P* < 0.01.

We next explored the molecular mechanism by which GLU‐mediated AHR activation facilitated mucin sulfation. GLU could benefit intestinal goblet cell homeostasis through modulating mucin O‐glycan biosynthesis and sulfur metabolism (Figure 2A,B). N‐acetylgalactosaminyltransferases (GALNT) family enzymes catalyze the initial step in mucin O‐glycan biosynthesis [26]. After analysis on transcriptome data, we found that DON impaired the expression of GLANT family proteins, and GLU could up‐regulate the expression of multiple GLANT proteins such as *GALNT2*, *GALNT7*, and *GALNT11* (Figure S12). As a key conjugation reaction, sulfation is essential for mucin stability and is facilitated by sulfotransferases [27]. Therefore, we then analyzed the gene expression of various enzymes involving sulfation. Notably, GLU treatment induced the expression of many sulfation‐associated enzymes, while DON challenge inhibited their expression (Figure 7A). Among these sulfation‐associated enzymes, only sulfotransferase *GAL3ST3* expression was elevated by GLU under both homeostasis conditions and DON challenge (Figure 7A,B). Western blot assay also showed that GLU administration effectively restored GAL3ST3 expression under DON challenge (Figure 7C). We then utilized an immunostaining assay to detect GAL3ST3 expression in porcine ileum. GAL3ST3 was mainly expressed in the intestinal villi epithelium (especially in goblet cells, black arrowheads) rather than crypt epithelium (Figure 7D), suggesting that villi‐restricted sulfomucin enrichment is associated with the differential expression of GAL3ST3 along the villi‐crypt axis. DON challenge reduced GAL3ST3 expression, and additional GLU administration significantly enhanced GAL3ST3 expression in porcine ileum (Figure 7E,F). Correlation analysis revealed that sulfomucin abundance was positively correlated with relative *GAL3ST3* expression in porcine ileum (Figure 7G). Besides, GLU‐associated LA and IAA could motivate GAL3ST3 expression in the ileum as well (Figure S7D–F).

To validate whether AHR signaling was of great importance to GAL3ST3 expression induced by GLU, we utilized AHR antagonist CH‐223191, together with DON and GLU, to treat IPEC‐J2 cells and porcine intestinal organoids. CH‐223191 administration successfully suppressed AHR signaling activity in vitro (Figure 7H). Notably, immunostaining and qPCR analyses showed that the promotive effect of GLU on GAL3ST3 expression was reversed by CH‐223191 (Figure 7I,J), suggesting that AHR signaling is vital for GLU‐mediated GAL3ST3 expression.

In *AHR* knockout mice, *GAL3ST3* mRNA expression was significantly reduced (Figure 7K). To investigate whether AHR could transcriptionally manipulate *GAL3ST3* expression, we constructed an AHR expression plasmid and a luciferase reporter plasmid containing the porcine *GAL3ST3* promoter to conduct a dual‐luciferase assay. AHR overexpression significantly increased the activity of the *GAL3ST3* promoter (Figure 7L). We then analyzed the potential binding sites of AHR on porcine *GAL3ST3* promoter with the Jasper database and noticed the presence of xenobiotic response elements (XRE, GCGTG), the AHR binding motif [28], in porcine *GAL3ST3* promoter. When the XRE in the *GAL3ST3* promoter was mutated or deleted, the activation effect of AHR on *GAL3ST3* promoter activity was vanished (Figure 7M). In addition, the CUT&RUN‐qPCR assay in IPEC‐J2 cells and porcine small intestinal epithelial cells revealed that AHR could directly bind to the promoter of porcine *GAL3ST3*, and GLU administration effectively strengthened their binding (Figure 7N,O). We then utilized *GAL3ST3* knockout mice (*GAL3ST3^−/−^*) to validate the necessity of GAL3ST3 in GLU‐mediated mucin sulfation and intestinal protection (Figure S13A,B). *GAL3ST3* knockout aggravated DON‐induced intestinal injury and sulfomucin loss, and GLU failed to alleviate DON‐induced intestinal injury and increase sulfomucin abundance in *GAL3ST3^−/−^* mice (Figure S13C–E), indicating that GAL3ST3 is of great importance for GLU‐mediated mucin sulfation and intestinal protection. To explore whether GLU metabolite such as glucuronic acid could promote AHR‐mediated GAL3ST3 expression, we treated IPEC‐J2 cells with glucuronic acid for 24 h. The result showed that glucuronic acid had no effect on the expression of *CYP1A1* and *GAL3ST3* (Figure S14). In conclusion, the above results suggest that GLU activates AHR‐mediated transcriptional regulation to promote sulfotransferase GAL3ST3 expression, thus facilitating mucin sulfation and intestinal protection.

KEGG analysis revealed that GLU also affected the TGF‐β signaling pathway (Figure 2A,B), and the AHR/Nrf2 signaling pathway has been reported to be protective against murine colitis [29]. To investigate their crosstalk with the AHR/GAL3ST3 signaling pathway, we treated mice with TGF‐β inhibitor SB431542 or Nrf2 inhibitor ML385 on the basis of GLU and DON administration (Figure S15A). Both SB431542 and ML385 treatments had no effect on GLU‐mediated *CYP1A1* and *GAL3ST3* expression (Figure S15B,C). In addition, under TGF‐β or Nrf2 inhibition, GLU still could attenuate DON‐induced intestinal injury (Figure S15D,E) and promote mucin sulfation (Figure S15F). These findings suggest that GLU facilitates AHR/GAL3ST3‐mediated mucin sulfation and intestinal protection independently of TGF‐β and Nrf2 signaling.

## 3 Discussion

The prevalence of DON in food and feed poses a serious threat to the health of humans and livestock [1]. As the first line of defense, the gut plays a crucial role in resisting DON toxicity. In the gut, the transcriptional similarity between pigs and humans is dramatically higher than that between mice and humans [30], suggesting that pigs represent a more appropriate model for studying human gut biology study. Therefore, in this work, we employed a piglet model to study the protective effect of GLU on DON‐induced intestinal injury. GLU is a natural metabolite of glucose and exerts potent immunoregulatory properties [14]. Previous studies have reported that GLU can ameliorate ochratoxin A‐induced oxidative stress and apoptosis [15]. In this study, we provide multiple pieces of evidence demonstrating that GLU attenuates DON‐induced intestinal injury by effectively promoting mucin sulfation.

As an essential part of intestinal innate immunity, goblet cells secrete abundant mucins to maintain mucus integrity, thus improving intestinal homeostasis [7]. Goblet cell dysfunction is implicated in many intestinal diseases such as IBD and pathogen infections [9]. Critically, sulfation confers resistance to bacterial degradation upon goblet cell‐derived mucins within the intestinal lumen [11]. In intestinal disorders such as diarrhea and colitis, sulfomucin abundance is markedly diminished, underscoring their vital role in reinforcing the intestinal barrier [25, 31]. Herein, we found that DON not only reduced goblet cell number as we previously reported [12], but also lowered sulfomucin abundance in the ileum. The significant correlation between mucin sulfation and intestinal injury strongly suggests that this modification could be a novel target for alleviating DON‐mediated intestinal toxicity. Using RNA sequencing analysis, we noticed that GLU could improve mucin biosynthesis and sulfation in the gut epithelium with or without DON challenge, strongly suggesting its potential efficacy in ameliorating intestinal disorders. However, DON and GLU affected goblet cell number in villus, but not in the crypts, which could be attributed to the fact that intestinal goblet cells in different locations have different functions [32]. Mucus from villus goblet cells protects the intestinal epithelium and permits small molecule passage, whereas the denser mucus from crypt goblet cells provides a specialized niche for intestinal stem cell protection [32]. Consequently, villus goblet cells could be more susceptible to DON or GLU modulation than their crypt counterparts, explaining the unaltered goblet cell counts observed in the crypts. We have demonstrated that GLU alleviates weaned stress‐induced intestinal injury via the Nrf2/Akt/FOXO1 Pathway [16]. In this work, we further demonstrate that GLU attenuates DON‐induced intestinal injury by targeting mucin sulfation. The modulatory effects of GLU on mucin homeostasis also reveal its therapeutic potential for human intestinal disorders such as IBD and pathogen infection, which needs to be investigated in our future work.

Notably, GLU supplementation reduced serum LPS level but had no significant effect on serum iFABP level. Within the small intestine, goblet cell density is highest in the ileum [7]. The beneficial effects of GLU on ileal goblet cells could protect enterocytes from DON‐induced injury as demonstrated by H&E staining. However, iFABP expression is highest in the jejunum, where goblet cell density is lower than in the ileum [33]. We proposed that the lower goblet cell density in the jejunum weakened the protective effect of GLU on jejunal health, potentially resulting in insufficient amelioration of jejunal injury. Consequently, the unchanged serum iFABP level following GLU treatment may be attributed to the combined effect of higher iFABP expression and lower goblet cell density in the jejunum compared to the ileum. Microbial density increases from the duodenum to the ileum, suggesting that ileal barrier function plays a more critical role than jejunal barrier function in determining serum LPS levels. Therefore, the reduced serum LPS level could reflect the improved intestinal barrier function in the ileum rather than the jejunum.

A review of the literature revealed no prior studies administering GLU to mouse models. To address this gap, we conducted an experiment to determine an appropriate GLU dosage in mice. Both low‐dose (40 mg/kg) and high‐dose (80 mg/kg) GLU promoted mucin sulfation in mice without affecting goblet cell number, indicating a key conceptual point that mucin sulfation could be regulated independently of goblet cell abundance. Unexpectedly, although no overt intestinal injury was observed, high‐dose GLU increased *TNF‐α* mRNA level in the ileum, indicating that this dosage could be excessive and pro‐inflammatory in mice. This aligns with our previous finding that high‐dose GLU is toxic to IPEC‐J2 cells [16]. The pro‐inflammatory effect of high‐dose GLU may attributed to its ability for AHR activation since AHR signaling must be tightly regulated to avoid oxidative stress [34]. Excessive AHR activation up‐regulates the expression of CYP1A1, a monooxygenase that generates reactive oxygen species (ROS), to trigger oxidative stress [34], which could induce intestinal inflammation. In this work, high‐dose GLU may over‐activate AHR to trigger CYP1A1 expression and oxidative stress, eventually boosting *TNF‐α* expression. Therefore, the optimal dose of GLU should balance AHR activation for mucosal protection while avoiding pro‐inflammatory effect.

It is generally accepted that goblet cell impairment leads to disorders in intestinal microbiota [35]. We found that the DON challenge induced bacterial invasion and altered the structure and composition of intestinal microbiota. Consistent with the previous studies [36, 37], DON challenge reduced the abundance of *Lactobacillus* spp. in the ileum. Notably, GLU treatment increased the abundance of several *Lactobacillus* spp. such as *L. amylovorus* and *L. reuteri*. Conversely, the intestinal microbiota can regulate goblet cell homeostasis [38]. Microbiota transplantation and co‐housing are established methods for investigating the functional role of the intestinal microbiota [39, 40]. Using these methods, we demonstrated that GLU‐associated intestinal microbiota contribute to mucin sulfation and the alleviation of DON‐induced intestinal injury. However, because IMT transfers a microbial community in porcine ileum, *L. amylovorus* could be identified as a major contributor rather than the sole driver facilitating mucin sulfation. Actually, *L. reuteri* abundance was elevated by GLU, which has been proved to possess the potential of promoting mucin sulfation [25]. Among various GLU‐modulated intestinal bacteria, *Lactobacillus spp*. exerts probiotic effects via its metabolites [41]. GLU‐induced changes in luminal metabolites were associated with tryptophan metabolism, a pathway facilitated by *L. amylovorus* and *L. reuteri* [23, 24]. In the gut, tryptophan is metabolized primarily through three pathways: the kynurenine, 5‐hydroxytryptamine, and indole pathways [42]. The indole pathway is mediated by the intestinal microbiota, which converts tryptophan into various indole derivatives [42]. A key metabolite of this pathway, IAA, can improve gut health by strengthening the intestinal barrier and immune response [43]. Previous work has shown that IAA administration increases sulfomucin levels in the colon to ameliorate colitis [25]. Similarly, in this work, we identified *L. amylovorus* and it‐derived IAA as key triggers for mucin sulfation in the ileum. Multiple *Lactobacillus spp*., including *Lactobacillus plantarum* [44] and *Lactobacillus paracasei* [45], can effectively detoxify DON through various mechanisms such as directly binding to DON and bio‐transforming DON into other products. However, whether GLU‐enriched *L. amylovorus* or *L. reuteri* could detoxify DON remains unclear, which is worth exploring in our future work. Following microbiota removal by ABX administration, GLU itself remained capable of facilitating mucin sulfation and ameliorating DON‐induced intestinal injury. Actually, in addition to GLU, other compounds such as baicalin also exert protective effects on intestinal disorders via both microbiota‐dependent and ‐independent manners. Specifically, both baicalin itself and baicalin‐associated *L. amylovorus* can suppress M1 macrophage polarization and Th17 cell differentiation to ameliorate pathogenic *Escherichia coli*‐induced intestinal inflammation [46].

AHR signaling plays a vital role in maintaining intestinal homeostasis. In IPEC‐J2 cells, AHR activation protects against DON‐induced inflammation via repressing TNF‐α/NF‐κB/MLCK signaling pathway [47]. In the present study, both GLU‐associated gut microbiota and GLU itself activated AHR signaling. IAA is a known ligand of AHR [25]. Using molecular docking and molecular dynamics simulations revealed that GLU also acted as a ligand for porcine AHR. Although our study demonstrates that GLU activates AHR through both direct binding and microbiota‐derived IAA, the relative contribution of each pathway remains to be fully quantified. Future studies employing competitive ligand‐binding assays or AHR mutant models will help elucidate the proportional roles of these dual activation mechanisms. As a transcription factor, AHR promotes *Atoh1* expression to facilitate intestinal goblet cell differentiation [48], which explains our finding that GLU boosted various types of N‐glycan biosynthesis and mucin type O‐glycan biosynthesis. Notably, GLU exerted a more robust effect on mucin sulfation than mucin biosynthesis since GLU administration contribute to a greater increase in sulfomucin area than goblet cell number. AHR‐mediated mucin sulfation has been demonstrated to be protective against dextran sulphate sodium‐induced colitis [25]. Similarly, in this study, AHR deficiency induced by gene editing or drug administration abolished the beneficial effects of GLU on mucin sulfation and against DON‐induced intestinal injury. However, AHR signaling broadly regulates immune homeostasis and barrier function in the gut. To determine whether the intestinal protective effect of GLU was specifically mediated through mucin sulfation, we constructed *GAL3ST3^−/^* ^−^ mice and found that GLU failed to alleviate DON‐induced intestinal injury and increase sulfomucin abundance in *GAL3ST3^−/−^* mice, strengthening the mechanistic specificity of GLU.

Sulfation renders mucins resistant to bacterial degradation [49]. According to the RNA sequencing result, DON dramatically down‐regulated the expression of many mucin sulfation‐associated enzymes, and GLU up‐regulated the expression of most mucin sulfation‐associated enzymes under homeostasis. Previous study has shown that IAA‐mediated AHR activation promotes *PAPSS2* and *SLC35B3* expression at the transcriptional level to boost mucin sulfation [25]. However, in our work, GLU did not affect *PAPSS2* or *SLC35B3* expression but selectively elevated the expression of the sulfotransferase *GAL3ST3* under DON challenge. This discrepancy could reflect ligand‐selective AHR activation since GLU exhibited a specific AHR‐binding pattern distinct from IAA. GLU‐ induced alterations in AHR conformation may preferentially promote *GAL3ST3* transcription. In addition, chromatin compaction or histone modifications at the *PAPSS2* and *SLC35B3* promoters in porcine intestinal epithelial cells may limit AHR binding, thus abolishing AHR‐mediated activation.

This study also has some limitations regarding experimental design and mechanistic exploration. In addition to CD163, more macrophage markers should be used to evaluate the macrophage recruitment in the ileum. Since CD163 has been utilized as a macrophage marker in piglets [50], herein, the reduced expression of CD163 by GLU could represent decreased inflammatory recruitment. Although *L. amylovorus* abundance positively correlates with IAA levels and its administration increases IAA, these data do not directly demonstrate that *L. amylovorus* is the primary or exclusive source of IAA in vivo. In our future work, we need to identify and delete IAA‐producing genes in *L. amylovorus* and then treated mice with the gene‐deleted *L. amylovorus* to observe the protective effect. The in vivo metabolic stability (degradation rate, absorption efficiency, and metabolites of GLU) of GLU was not evaluated in this study, which needs to be explored with isotope‐labeled GLU in our future work. The current experimental design does not distinguish whether GLU functions preventively or therapeutically since GLU is administered simultaneously with DON. In our opinion, GLU could be acting preventively against DON‐induced intestinal injury since GLU could facilitate mucin sulfation and ZO‐1 expression without DON challenge, supporting a preventive role against subsequent insult. An intestinal repair model (such as the recovery period after 7‐day dextran sulfate sodium treatment) could be used to investigate whether GLU could promote intestinal repair after injury initiation in our future work. While this study demonstrates the efficacy and mechanistic basis of GLU in alleviating DON‐induced intestinal injury, several translational aspects require further investigation. These include the stability of GLU during feed/food processing and storage, its potential interactions with other dietary components, and the long‐term safety of GLU supplementation, particularly regarding hepatic and renal functions. Our future studies will focus on these practical concerns to facilitate the potential application of GLU as a feed additive or nutritional supplementation.

**FIGURE 8:**
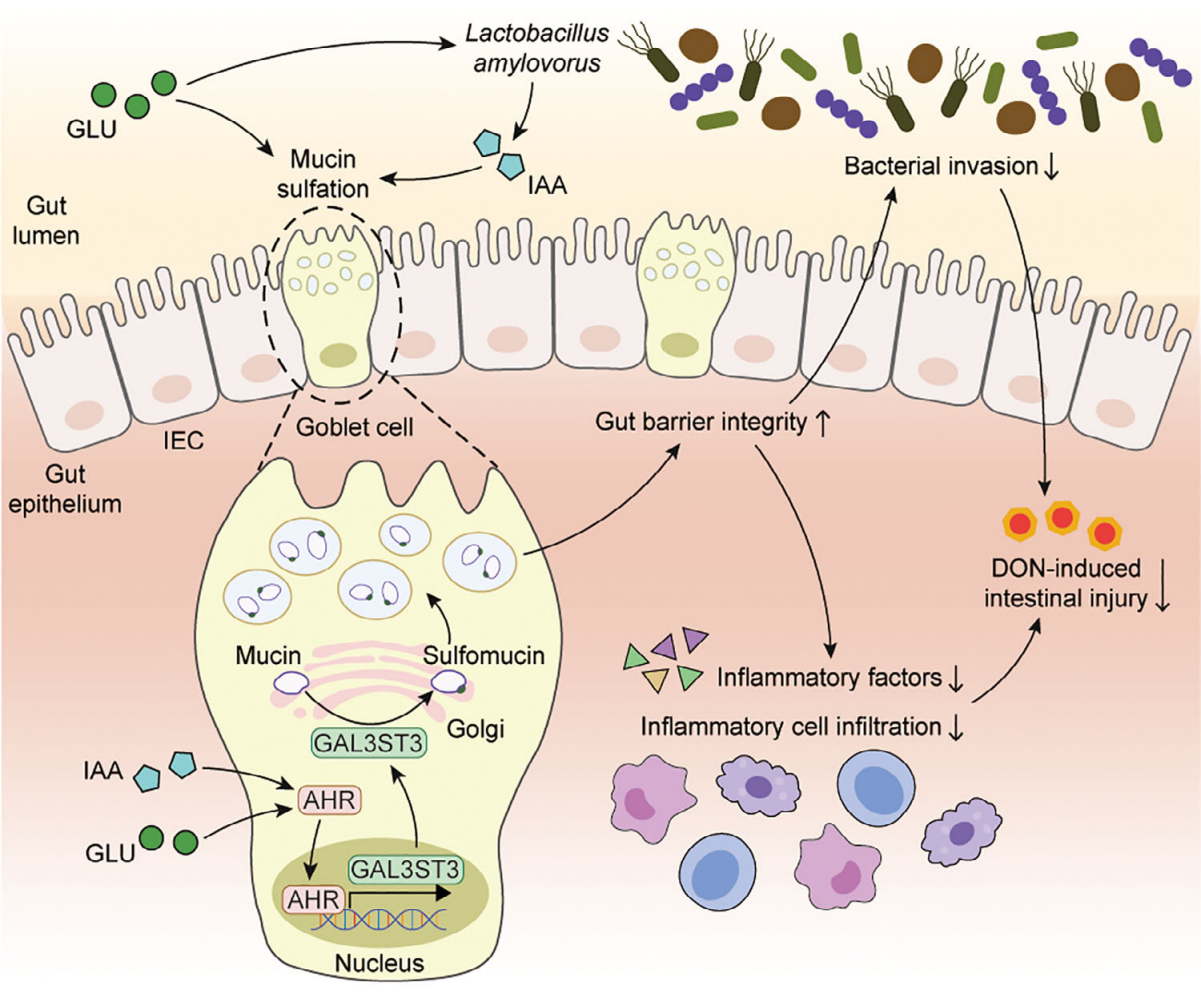
Proposed model for GLU‐mediated mucin sulfation and intestinal protection. GLU as a natural metabolite of glucose increases *Lactobacillus amylovorus* abundance and luminal IAA level to activate AHR signaling. In addition, GLU itself can directly elevate AHR signaling activity independently of microbiota and IAA. AHR activation by GLU transcriptionally boosts sulfotransferase *GAL3ST3* expression to promote mucin sulfation, which strengths the gut barrier integrity and eventually attenuates DON‐induced intestinal injury.

In summary, this work systematically explores the mechanisms by which GLU attenuates DON‐induced intestinal injury through both microbiota‐dependent and ‐independent pathways. On the one hand, GLU increases *L. amylovorus* abundance and luminal IAA level to activate AHR signaling. On the other hand, GLU itself can directly elevate AHR signaling activity independently of the microbiota and IAA. AHR activation by GLU transcriptionally facilitates sulfotransferase *GAL3ST3* expression to promote mucin sulfation, thus alleviating DON‐induced intestinal injury (Figure 8). Our findings identify GLU as a promising nutritional intervention or therapeutic agent to counteract the hazard of DON contamination on gut health, highlighting AHR‐driven mucin sulfation as a critical host defense mechanism against DON‐induced intestinal injury.

## 4 Experimental Section

### 4.1 Animal Experiments

All experimental procedures and animal care protocols received approval from the Animal Care and Use Committee of the Guangdong Academy of Agricultural Sciences (approval number: 2024011). For the piglet experiment, a total of 24 weaned piglets (Duroc × Landrace × Yorkshire; 21 days old; half male and half female) were randomly allocated to four dietary treatments (*n* = 6). The piglets in the control group (CON) were fed with a basal diet, and the piglets in other groups were fed the basal diet supplemented with 200 mg/kg GLU (GLU), 2.4 mg/kg DON (DON), or 200 mg/kg GLU plus 2.4 mg/kg DON (GLU+DON) for a 5‐week feeding trial. The dosage of GLU was determined according to our previous study [16], which was proved physiologically achievable and safe to piglets. The 2.4 mg/kg DON reflected moderate‐to‐high contamination events and was commonly used to induce intestinal barrier dysfunction in piglets [51]. GLU (≥98%) was supplied by Zhucheng Haotian Pharmaceutical Co., Ltd., Zhucheng, Shandong, China. DON was prepared with *Fusarium graminearum* and corn based on methods in a previous study [52]. The DON concentration in final diets of different groups was detected with a commercial Deoxynivalenol ELISA Kit (HWEKT‐030, Pribolab) according to the manufacturer's instructions and is shown in Table S1. After the experiment, the piglets were humanely euthanized, and the serum and ileal samples were harvested for further analyses.

Six to eight‐week‐old WT C57BL/6J mice were obtained from the Southern Medical University Laboratory Animal Center (Guangzhou, China). *AHR* knockout and *GAL3ST3* knockout mice (C57BL/6J background) were purchased from Cyagen US Inc (Guangzhou, China). The mice were housed under controlled conditions of 22°C ± 1°C, 70% ± 5% humidity, and a 12‐hour light/dark cycle. All mice were allowed to have food and water ad libitum.

For the IMT experiment, experimental procedures were conducted in accordance with the protocols described by Zhang et al. [39] and Yang et al. [53] with some modifications. In brief, fresh ileal contents were collected from piglets in the CON group and the GLU group, respectively. Sterile PBS solution containing 10% glycerol was added to resuspend the ileal contents (40 mL PBS: 1 g contents) in an anaerobic incubator. Then, the liquid suspension was vortexed and filtered through a 70 µm strainer to remove large particulates. The filtered mixture was centrifuged at 200 × g for 5 min, and the supernatant was collected and stored at −80°C for gavage. A total of 10 recipient C57BL/6J mice were randomly divided into IMT‐CON and IMT‐GLU groups (*n* = 5). All mice were administered an ABX cocktail with ampicillin (1 g/L), metronidazole (1 g/L), neomycin sulfate (1 g/L), vancomycin (0.5 g/L), sucrose (0.1%) through drinking water for 2 weeks. After ABX treatment, mice were given sterile water for two days. The above microbiota‐removing procedures were conducted according to the previous study [25]. Subsequently, mice received 200 µL of thawed suspension from CON piglets or GLU piglets via oral gavage for 14 consecutive days.

For the murine GLU experiment, a total of 13 mice were randomly divided into three groups: CON group (*n* = 4), low GLU group (GLU, *n* = 5, 40 mg/kg), and high GLU group (HGLU, *n* = 4, 80 mg/kg). Mice received daily oral gavage of PBS or GLU (40 or 80 mg/kg, 851 450, Sigma‐Aldrich) for 10 consecutive days.

For the co‐housing and GLU treatment experiment, a total of 24 mice were randomly divided into four groups (*n* = 6): CON group, GLU group, CON‐co‐housing group, and GLU‐co‐housing group. The mice in the CON group and GLU group were kept in different cages, and the mice in the CON‐co‐housing group and GLU‐co‐housing group were kept in the same cage as described by the previous study [54]. The mice in the GLU group and GLU‐co‐housing group were gavaged with 40 mg/kg GLU every two days for 30 days.

For the co‐housing, GLU treatment, and DON challenge experiment, a total of 18 mice were randomly divided into three groups (*n* = 6): DON group, DON‐co‐housing group, and GLU+DON‐co‐housing group. The mice in the GLU+DON‐co‐housing group were gavaged with 40 mg/kg GLU every two days for 30 days. The mice in the DON‐co‐housing group and the GLU+DON‐co‐housing group were co‐housed for the same 30 days. Then, the mice in the DON group and the DON‐co‐housing group received an oral gavage of 2 mg/kg DON (D0156, Sigma‐Aldrich) every day for 1 week. The dosage of DON was determined according to our previous study [13].

For the IAA experiment, a total of 12 mice were randomly divided into two groups (*n* = 6): the CON group and the IAA group. The mice in the IAA group were administered IAA (20 mg/kg, I3 750, Sigma‐Aldrich) for 10 consecutive days. The dosage of IAA was determined based on the previous study [25].

For the *L. amylovorus* experiment, *L. amylovorus* was purchased from BeNa Culture Collection (BNCC342646). A total of 12 mice were randomly divided into two groups (*n* = 6): the CON group and the LA group. Before *L. amylovorus* treatment, all mice were administered an ABX cocktail as described above. Then, the mice in the LA group received 10^9^ CFU *L. amylovorus* three times a week for two weeks.

For the *L. amylovorus* treatment and DON challenge experiment, a total of 19 mice were randomly divided into two groups (*n* = 6): CON group (*n* = 6), DON group (*n* = 7), and LA+DON group (*n* = 6). Mice received an ABX cocktail for 2 weeks and *L. amylovorus* treatment for another 2 weeks as described before. During the last week of *L. amylovorus*, the mice in the DON group (*n* = 7), and the LA+DON group (*n* = 6) were given 2 mg/kg DON via oral gavage every day.

For the ABX, GLU treatment, and DON challenge experiment, after ABX administration, mice received 40 mg/kg GLU for 14 consecutive days. At day 8, mice were challenged with 2 mg/kg DON for 7 consecutive days. To validate the microbiota removal by ABX, fresh feces were weighted and homogenized with sterile PBS. Fecal suspension was diluted and plated on LB agar. After 12 h, the colony numbers on the LB agar were counted, and the total bacterial load was calculated.

For inhibitor treatments, mice were treated with 10 mg/kg CH‐223191 (S7711, Selleck, intraperitoneal injection), 16 mg/kg SB431542 (S1067, Selleck, intraperitoneal injection), or 30 mg/kg ML385 (S8790, Selleck, gavage) every two days to inhibit AHR, TGF‐β, and Nrf2 signaling, respectively. The dosages of these inhibitors were determined according to the previous study [55, 56, 57].

### 4.2 IPEC‐J2 Cell Culture and Treatment

IPEC‐J2 cells obtained from Procell Life Science & Technology Co., Ltd (CL‐0973) were seeded in 24‐well plates with a density of 5 × 10^5^ cells per well and cultured in Dulbecco's Modified Eagle Medium/Nutrient Mixture F‐12 (DMEM/F‐12, 11320033, Gibco) supplemented with 10% fetal bovine serum (FBS, 10270106, Gibco) and 1% penicillin‐streptomycin (15140122, Gibco) at 37°C in a 5% CO_2_ atmosphere. IPEC‐J2 cells were divided into four groups: CON group, GLU group, DON group, and GLU+DON group. In accordance with our previous work, the dosages of GLU and DON were 1000 µg/mL and 500 ng/mL, respectively [16, 58]. The dosage of CH‐223191 was based on the previous study [25]. GLU, DON, or CH‐223191 was added into the medium at the same time. After 24 h, IPEC‐J2 cells were harvested for subsequent analysis. For glucuronic acid treatment, 500 or 1000 µg/mL glucuronic acid (HY‐N6612, MCE) was used to treat IPEC‐J2 cells for 24 h.

### 4.3 Histology, Immunohistochemistry, and Immunofluorescence Staining

Ileal tissues, intestinal organoids, or IPEC‐J2 cells were fixed in 4% paraformaldehyde (G1101, Servicebio). Then, ileal tissues or intestinal organoids were embedded in paraffin and cut to 5 µm for subsequent analysis. The procedures of H&E staining, alcian blue/periodic acid‐Schiff (AB/PAS) staining, immunohistochemistry, and immunofluorescence were conducted according to the methods in previous studies [53, 59, 60]. Epithelial damage score and inflammation score were conducted in a blinded manner according to the previous study [46]. For high iron diamine/alcian blue (HID/AB) staining, the paraffin sections were stained with an HID/AB mucins stain kit (G2070, Solarbio), and the brown–purple to brown–black dots could be considered as sulfomucins in epithelium. For MALII lectin staining, the experimental procedures were performed with biotinylated MALII (B‐1265‐1, Vector Laboratories, 1:200) and YSFluor 488‐conjugated streptavidin (35103ES60, Yeasen Biotechnology, 5 µg/mL) according to the methods described in a previous study [17]. The primary antibodies for immunohistochemistry and immunofluorescence: anti‐Claudin‐1 (28674‐1‐AP, Proteintech, 1:500), anti‐CD163 (MCA2311A647, BIO‐RAD, 1:200), anti‐MUC2 (27675‐1‐AP, Proteintech, 1:250), anti‐ZO‐1 (21773‐1‐AP, Proteintech, 1:200), anti‐F4/80 (29414‐1‐AP, Proteintech, 1:250), anti‐AHR (AF6278, Affinity Biosciences, 1:200), anti‐CYP1A1 (13241‐1‐AP, Proteintech, 1:200), anti‐GAL3ST3 (24851‐1‐AP, Proteintech, 1:200). Secondary antibodies: Alexa Fluor 488 AffiniPure Goat Anti‐Rabbit IgG (111‐545‐003, Jackson ImmunoResearch) and Cy3 AffiniPure Goat Anti‐Rabbit IgG (111‐165‐003, Jackson ImmunoResearch). The sections were examined with Olympus IX83 microscope with objectives (10×, NA = 0.30; 20×, NA = 0.65; 40×, NA = 0.95; 100×, NA = 1.40). The sulfomucin area and immunostaining positive areas were quantified using Image J software.

### 4.4 Enzyme‐Linked Immunosorbent Assay (ELISA)

LPS and iFABP levels in serum were detected using the LPS ELISA kit (MM‐0634M2, Jiangsu Meimian Industrial Co., Ltd.) and the porcine iFABP ELISA kit (MM‐261802, Jiangsu Meimian Industrial Co., Ltd.) based on the manufacturer's instructions. IAA levels in ileal contents were examined with the IAA Chemiluminescent Immunoassay Kit (abx190011, Abbexa).

### 4.5 Quantitative Real‐Time PCR

Total RNA from ileal tissues or IPEC‐J2 cells was extracted using RNA isolater Total RNA Extraction Reagent (R401‐01, Vazyme) and reversely transcribed into cDNA with ABScript Neo RT Master Mix for qPCR with gDNA Remover (RK20433, Abclonal). Then, mRNA levels were examined by qPCR analysis with ChamQ SYBR qPCR Master Mix (Q321, Vazyme). Relative mRNA expression was normalized to glyceraldehyde‐3‐phosphate dehydrogenase (GAPDH) mRNA with the “2^−ΔΔCt^” method. The primers in this study are listed in Table S2.

As for the *L. amylovorus* quantification, total DNA in feces was extracted using the DNA Stool Kit (51604, QIAGEN). qPCR was then conducted to detect the relative abundance of *L. amylovorus* in feces as described above. The primers of *L. amylovorus* and the universal bacterial 16S gene are listed in Table S2.

### 4.6 Transcriptome Analysis

Porcine intestinal epithelial cell line IPEC‐J2 cells were washed with PBS and collected for RNA sequencing. RNA extraction, quality control, library preparation, and sequencing were conducted by Shanghai Majorbio Bio‐Pharm Technology Co. Ltd. (Shanghai, China). The criteria of high‐quality RNA samples were as following: OD260/280 = 1.8–2.2, OD260/230 ≥ 2, RQN ≥ 6.5, 28S:18S ≥ 1, and total RNA amount > 1 µg. Identification of DEGs was performed using DESeq2 under the condition of *p* < 0.05 and a fold change ≥ 2. KEGG pathway analysis was conducted to identify which DEGs were significantly enriched in metabolic pathways at Bonferroni‐corrected *p* < 0.05 compared with the whole‐transcriptome background. KEGG pathway analysis was carried out using the Majorbio Cloud platform (https://cloud.majorbio.com). The raw RNA sequencing data were uploaded to the NCBI sequence read archive (SRA) database for storage (accession number: PRJNA1312445).

### 4.7 FISH Analysis

Porcine ileal sections were deparaffinized and rehydrated, subsequently hybridized with a universal bacterial probe, which was in accordance with the method described previously [61]. The primer for Cy3‐labeled universal bacterial probe was 5’‐CY3‐GCTGCCTCCCGTAGGAGT‐3’.

### 4.8 rRNA Sequencing Analysis

Porcine or murine ileal contents were collected, and genomic DNA was extracted using the QIAamp DNA Stool Mini Kit (51604, QIAGEN) following the manufacturer's instructions. After PCR amplification of The V3‐V4 region of the bacterial 16S rRNA gene was amplified using primers 338F (5'‐ACTCCTACGGGAGGCAGCAG‐3') and 806R (5'‐ GGACTACHV GGGTWTCTAAT‐3') via PCR. Purified amplicons were pooled in equimolar amounts and paired‐end sequenced on an Illumina Nextseq2000 platform according to the standard protocols by Majorbio Bio‐Pharm Technology Co. Ltd. (Shanghai, China). The raw 16S rRNA sequencing reads were deposited into the NCBI SRA database for storage (accession number: PRJNA1312483). Bioinformatic analysis of the ileal microbiota was performed using the Majorbio Cloud platform (https://cloud.majorbio.com). For the co‐occurrence network diagram, differences in abundance were analyzed by Spearman's rank correlation analysis. Co‐occurrence networks were constructed using data with correlation coefficients |*r*‐value| > 0.8 and *p *< 0.05. CytoScape software (v3.10.0) was used for visualization and network analysis.

### 4.9 Metabolomics Analysis

Non‐targeted metabolomics analysis was performed on porcine ileal contents. The LC‐MS/MS analysis was conducted by Majorbio Bio‐Pharm Technology Co. Ltd. (Shanghai, China). After preparation, samples were analyzed using a UHPLC‐Q Exactive HF‐X system (Thermo, Waltham, USA) equipped with an ACQUITY HSS T3 column (Waters Corporation, Milford, USA). Raw data were imported into Progenesis QI (Waters Corporation, Milford, USA) to process preliminarily. The metabolites were determined by matching with metabolite public database such as HMDB (http://www.hmdb.ca/), Metlin (https://metlin.scripps.edu/), and the self‐compiled Majorbio database. The data matrix obtained by searching the database was uploaded to the Majorbio cloud platform (https://cloud.majorbio.com) for data analysis.

### 4.10 Isolation of Porcine Intestinal Crypts and Organoid Culture

Porcine small intestinal crypts were isolated and cultured to establish intestinal organoids, which was conducted according to the previous study [62]. In brief, after sacrifice, approximately 10 cm of small intestinal tissues were opened lengthwise and washed with cold Dulbecco's PBS (DPBS) to remove the contents. The mesentery and villi were removed from the intestinal tissues, and then the tissues were cut into 5 mm × 5 mm sections and washed with cold DPBS for 10 times. Then intestinal pieces were resuspended with Gentle Cell Dissociation Reagent (100‐0485; STEMCELL Technologies) and incubated for 25 min at room temperature. After removing the reagent, the intestinal pieces were washed with cold DPBS (containing 0.1% bovine serum albumin), and the supernatant was filtered with a 70 µm strainer. Subsequently, the crypts were collected through centrifuged at 290 × g for 5 min. Finally, the crypts were resuspended with Matrigel (356231, Corning) and plated in a 24‐well plate. After polymerization for 10 min at 37°C, the crypts were cultured in 500 µL complete IntestiCult Organoid Growth Medium (06010, STEMCELL Technologies) supplemented with 1% penicillin‐streptomycin. Organoid medium was changed every 3–4 days, and the intestinal organoids were passaged at a ratio of 1:2–1:4 when necessary. The dosages of IAA, GLU, and CH‐223191 applied to organoids were 100 µm, 1000 µg/mL, and 10 µm, respectively, based on the previous studies [16, 25].

### 4.11 IAA Production From *Lactobacillus amylovorus* In Vitro

*L. amylovorus* was cultured in MRS media supplemented with or without 3 mm tryptophan. After 24 h, the media were collected and centrifuged at 5000 rpm for 10 min. IAA levels in the supernatants were detected with the IAA Chemiluminescent Immunoassay Kit (abx190011, Abbexa).

### 4.12 Nuclear Protein Isolation

After treatment, IPEC‐J2 cells were washed with cold PBS and collected for nuclear protein isolation. Nuclear proteins were purified using the Nuclear and Cytoplasmic Protein Extraction Kit (P0027, Beyotime Biotechnology) according to the manufacturer's instructions.

### 4.13 Western Blot

The total protein was extracted with Radio Immunoprecipitation Assay lysis buffer (P0013B, Beyotime Biotechnology) supplemented with protease inhibitor cocktail. Then, purified protein was quantified using the Enhanced BCA Protein Assay Kit (P0010, Beyotime Biotechnology) according to the manufacturer's instructions. Proteins (20–40 µg) were separated by sodium dodecyl sulfate‐polyacrylamide gel electrophoresis followed by blotting on polyvinylidene fluoride (PVDF) membrane (ISEQ00010, Immobilon). After incubation with 5% nonfat milk (GC310001, Servicebio) for 2 h at room temperature to block non‐specific protein binding, the PVDF membrane was then incubated with the primary antibody and secondary antibodies. The primary antibodies included: anti‐Claudin‐1 (28674‐1‐AP, Proteintech, 1:1000), anti‐AHR (AF6278, Affinity Biosciences, 1:1000), anti‐CYP1A1 (13241‐1‐AP, Proteintech, 1:1000), anti‐GAL3ST3 (24851‐1‐AP, Proteintech, 1:1000), anti‐Lamin B1 (12987‐1‐AP, Proteintech, 1:5000), and anti‐β‐actin (AC026, ABclonal, 1:100000). Second antibodies: Goat Anti‐Rabbit IgG HRP (S0001, Affinity Biosciences) and Goat Anti‐Mouse IgG HRP (S0002, Affinity Biosciences). Protein levels were quantified using the ImageJ software.

### 4.14 Molecular Docking and Molecular Dynamics Simulations

The molecular structures of GLU and IAA were obtained from the PubChem database (http://pubchem.ncbi.nlm.nih.gov/, Compound CID: 92283 and 802), and the molecular structure of porcine AHR was established with the AlphaFold3 tool. The molecular structures of both the protein and the small molecule were processed using AutoDock 1.5.6. Hydrogen atoms were added to the protein, and the small molecule ligand was prepared by adding hydrogens and defining torsional degrees of freedom. Subsequently, the coordinates of the docking box were determined. AutoDock Vina 1.1.2 software was used to process the structures of GLU and porcine AHR and conduct protein–ligand docking. The binding interactions were edited and visualized using PyMOL 2.6 software and Discovery Studio 2019 software.

In this study, molecular dynamics simulations were performed using GROMACS 2022, with force field parameters generated through the pdb2gmx tool within GROMACS. The GAFF2 force field topology file for the ligand was constructed based on its molecular structure using the sobtop_1.0 (dev3.1) software, and atomic partial charges were assigned via the RESP method to ensure a physically and chemically reasonable charge distribution. For the receptor protein, the AMBER14SB force field parameters were employed. The system was solvated using the TIP3P water model within a cubic water box extending 1 nm from the solute surface, ensuring adequate solvation and overall charge neutrality. To maintain electrical neutrality under simulated physiological conditions, Na^+^ and Cl^−^ ions were introduced into the system using the gmx_genion tool in GROMACS, with an ionic concentration of 0.15 m NaCl. Long‐range electrostatic interactions were treated using the Particle Mesh Ewald (PME) method with a cutoff distance of 1 nm. All force field parameters and PME‐related settings were optimized in accordance with GROMACS guidelines. Bond constraints were applied using the LINCS algorithm. Prior to production molecular dynamics simulations, the system underwent an energy minimization procedure. This consisted of 3000 steps of steepest descent optimization followed by 2000 steps of conjugate gradient minimization. The minimization was conducted in three sequential stages: first, with the solute constrained while water molecules were relaxed; second, with counterions constrained during energy minimization; and finally, with no constraints applied to the entire system. During the simulation, the temperature was maintained at 310 K using the Nosé–Hoover thermostat, and the pressure was kept at 1 bar via the Parrinello–Rahman barostat. The production run was performed under NPT (isothermal–isobaric) ensemble conditions for a duration of 100 ns with an integration time step of 2 fs. RMSD and HBond calculations were carried out using the gmx_rmsd and gmx_hbond tools in GROMACS, respectively.

### 4.15 DARTS Assay

DARTS assay was performed according to the reported protocol [63]. Briefly, cell lysates from HEK‐293T cells expressing AHR^WT^ or AHR^ARG‐366 MUT^ were centrifuged at 4°C and 12 000 g for 15 min, and the supernatants were collected for protein concentration examination by BCA assay. The supernatants containing the same protein amount (30 µg) were mixed with TNC buffer (50 mm Tris, 50 mm NaCl, and 10 mm CaCl_2_) and incubated with PBS or GLU at room temperature for 1 h. Then, the protein samples were digested with pronase (10165921001, Roche) for 30 min at 37°C. After digestion, protein samples were boiled for the subsequent Western blot analysis.

### 4.16 Dual‐Luciferase Reporter Assay

The sequence of the porcine *GAL3ST3* promoter fragment (2000 bp) was acquired from the Ensembl gene database (http://asia.ensembl.org/index.html) and chemically synthesized by Tsingke Biotech (Beijing, China). The wild type, mutation type, and deletion type of porcine *GAL3ST3* promoter fragments were ligated into the pGL3‐basic vector plasmid, which was constructed by Tsingke Biotech (Beijing, China). The porcine AHR expression plasmid was constructed by Bio‐Transduction Lab (Wuhan, China). HEK293T cells from Procell Life Science & Technology Co., Ltd (CL‐0005) were cultured in high‐glucose DMEM medium (11965092, Gibco) supplemented with 10% FBS (10270106, Gibco) and 1% penicillin‐streptomycin (15140122, Gibco) at 37°C in a 5% CO_2_ atmosphere. The expression plasmid and/or promoter plasmid was transfected into HEK293T cells together with Renilla luciferase reporter plasmid pTK (11557ES03, Yeasen Biotechnology) using Lipo8000 transfection reagent (C0533, Beyotime Biotechnology). The Dual Luciferase Reporter Assay Kit (DL101, Vazyme) was utilized to examine luciferase. Then, the activities of Firefly luciferase and Renilla luciferase were detected using a Synergy2 instrument (BioTek, USA).

### 4.17 CUT&RUN Assay

After PBS or GLU treatment for 24 h, IPEC‐J2 cells were washed with cold PBS and collected. Porcine small intestinal epithelial cells were isolated as described before [64]. CUT&RUN assay was performed to IPEC‐J2 cells and porcine small intestinal epithelial cells using Hyperactive pG‐MNase CUT&RUN Assay Kit (HD101, Vazyme) according to the manufacturer's instructions. The used antibodies included anti‐AHR antibody (83200, Cell Signaling Technology) and a rabbit control IgG (AC005, Abclonal). Then, qPCR was performed to detect target sequences. Primer sequence of porcine GAL3ST3 promoter for CUT&RUN‐qPCR is as listed: F‐TGGATTCCTGCTGGAGAGT, R‐GGAGGTAGAGACAGGTGTATG.

### 4.18 Statistical Analysis

All data were analyzed with GraphPad Prism software (v8.0.1) and Microsoft Excel (2021). For normally‐distributed data, unpaired Student's *t*‐test (two‐tailed) and one‐way ANOVA with Tukey's multiple comparisons test were used to examine the differences between groups. For abnormally distributed data, the Mann–Whitney *U*‐test (two‐tailed) and the Kruskal–Wallis test with Dunn's multiple comparison test were conducted to verify the differences between groups. Correlation analysis was conducted using Spearman's rank test correlation analysis suitable for ordinal or non‐normally distributed data. Data were expressed as the mean ± standard error of the mean (SEM). Differences were considered as significant at *P *< 0.05.

## Author Contributions

Study concept and design: Z.J., X.Y., Y.Q., and C.C. Experiment conducting and data analysis: C.C., J.T., J.H., and B.Z. Manuscript drafting and revision: C.C. and X.Y. Study supervision: Z.J., X.Y., L.W., and K.G.

## Funding

This work was financially supported by National Key R&D Program of China (2021YFD1300402), Guangdong Basic and Applied Basic Research Foundation (2025A1515012362), Modern Agricultural Industrial Technology System Innovation Team of Guangdong Province (2024CXTD14, 2024CXTD22), Special Fund for Scientific Innovation Strategy–construction of High‐Level Academy of Agriculture Science (R2023PY‐JG013, R2020PY‐JX007), earmarked fund for China Agriculture Research System (CARS‐35).

## Conflicts of Interest

The authors declare no conflict of interest.

## Supporting information




**Supporting file**: advs74499‐sup‐0001‐SuppMat.docx.

## Data Availability

The data that support the findings of this study are openly available in Sequence Read Archive (SRA) at 1312445, reference number 1312445.
