# Supplementary material for: Glucuronolactone Promotes Mucin Sulfation to Alleviate Deoxynivalenol‐Induced Intestinal Injury via Microbiota‐Dependent and ‐Independent AHR Activation
Source: Adv Sci (Weinh). 2026 Feb 23;13(25):e22912. doi: 10.1002/advs.202522912 (PMC13137803; doi:10.1002/advs.202522912)
Supplement: Supplementary file 1 — Supporting file: advs74499‐sup‐0001‐SuppMat.docx. [file ADVS-13-e22912-s001.docx]

**Table S1. Deoxynivalenol concentration in final diets of different groups**

| Group | CON | GLU | DON | GLU+DON |
| --- | --- | --- | --- | --- |
| Concentration  (mg/kg) | 0.56 | 0.57 | 2.41 | 2.43 |

The DON concentration in final diets of different groups was detected with a commercial Deoxynivalenol ELISA Kit (HWEKT-030, Pribolab) according to the manufacturer's instructions.

**Table S2. Primer sequences for qPCR**

| **Gene** | **Forward primer 5'-3'** | **Reverse primer 5'-3'** |
| --- | --- | --- |
| *pZO-1* | ACAGAGTCAGCTCAGCGAAC | GAACTGACTTGGCAGGCTCT |
| *pOcculudin* | CAGTGGTAACTTGGAGGCGT | CCGTCGTGTAGTCTGTCTCG |
| *pClaudin-1* | GGCCCTTACCTTTCGCCTGA | GCCTCAGGGCTTGGTGTTCT |
| *pTNF-α* | CCAATGGCAGAGTGGGTATG | TGAAGAGGACCTGGGAGTAG |
| *pIL-8* | TACGCATTCCACACCTTTC | GGCAGACCTCTTTTCCATT |
| *pIL-10* | CTGCATCCACTTCCCAACCA | CGGCATTACGTCTTCCAGGT |
| *pMuc2* | ACGCCATCCTGGGTGAGCT | ACGCTGCCGTCCGACTTGA |
| *pTff3* | GGGAAAAAGCTCCCTGGCTA | TCAAGGGTCACGGAAAGTGG |
| *pCYP1A1* | AGAAGTCCCGTAGGTGGAGA | GGGCTCAAAGTGGGCATGTA |
| *pCYP1B1* | AATAACGGGGGAAATTCCTG | CACCGAAACACAATGCAATC |
| *pGAL3ST3* | TAAGCCTCCTCATCCACCA | CCGTCTTGTGCGTCTTCA |
| *pGAPDH* | CGTCCCTGAGACACGATGGT | GCCTTGACTGTGCCGTGGAAT |
| *mTNF-α* | AAGCCTGTAGCCCACGTCGTA | AGGTACAACCCATCGGCTGG |
| *mIL-6* | TCCATCCAGTTGCCTTCTTG | TTCCACGATTTCCCAGAGAAC |
| *mIL-1β* | GATGATAACCTGCTGGTGTGTGA | TTTGTCGTTGCTTGGTTCTCC |
| *mIL-10* | GAGAGCTGCAGGGCCCTTTGC | CTCCCTGGTTTCTCTTCCCAAGACC |
| *mZO-1* | TCATCCCAAATAAGAACAGAGC | GAAGAACAACCCTTTCATAAGC |
| *mOcculudin* | CTTTGGCTACGGAGGTGGCTAT | CTTTGGCTGCTCTTGGGTCTG |
| *mClaudin-1* | AGAAGATGTGGATGGCTGTC | AATTCATACCTGGCATTGATGG |
| *mCYP1A1* | GAGGTTACTGGCTCTGGATAC | ATGAGGCTGTCTGTGATGTC |
| *mCYP1B1* | CACCACCTTCCGCAAGTT | GCACATCCTCCAAGTCCAG |
| *mGAL3ST3* | TTCTGCTGTTGGTGCTAGG | CCTGCTGTCTTGTGAGTCTT |
| *mGAPDH* | AACTTTGGCATTGTGGAAGG | GGATGCAGGGATGATGTTCT |
| *L.amylovorus* | CAAGCACGATTGGCAAGATG | ATTGGATTCCGCTTCTGTGG |
| *16S* | AGAGTTTGATCCTGGCTCAG | GGTTACCTTGTTACGACTT |

p, porcine; m, murine.


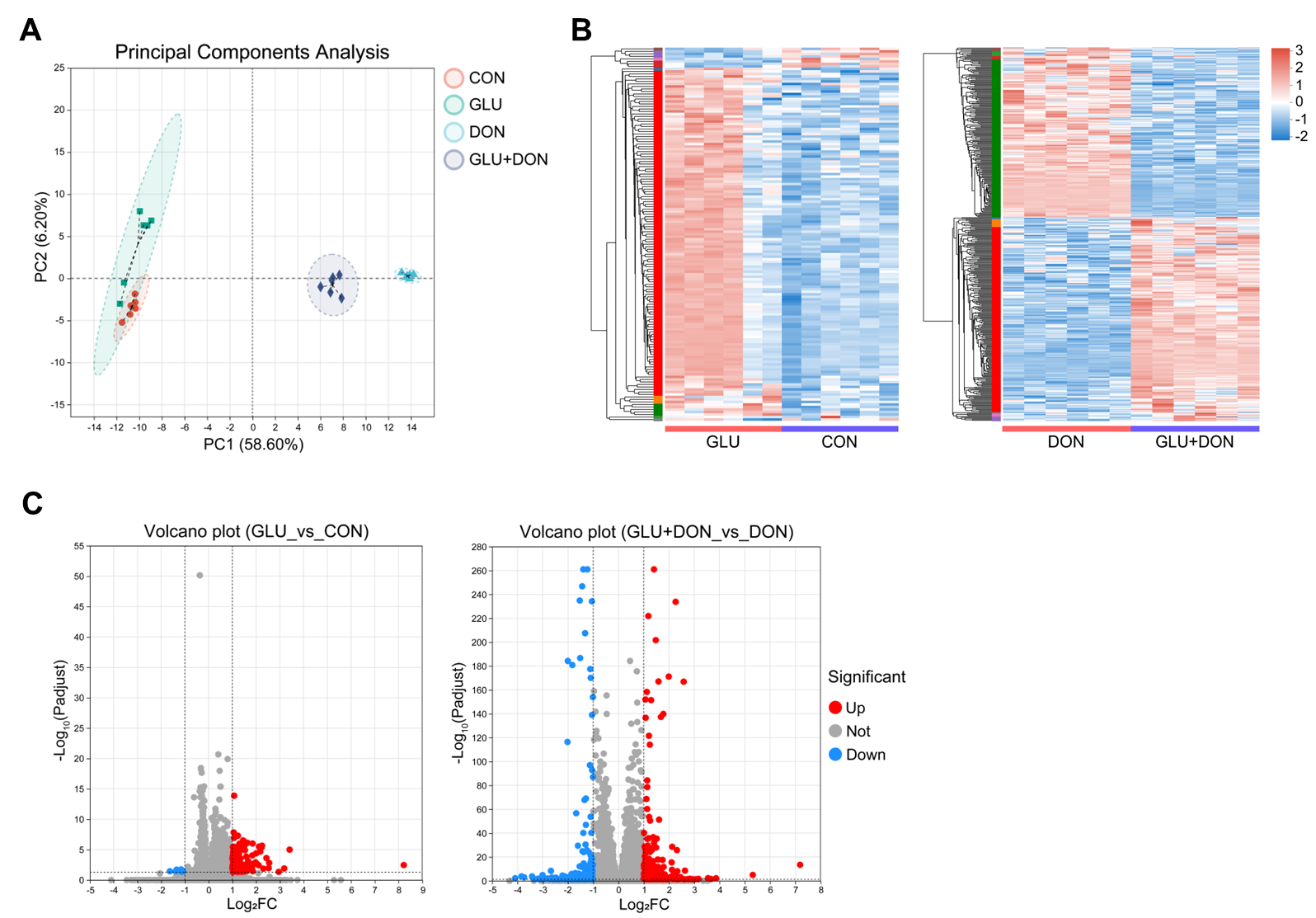


**Figure S1. Transcriptome of the intestinal epithelium of piglets.** (A) The PCA plot of transcriptome data from porcine intestinal epithelium. n = 6. (B) The cluster analysis of transcriptome data (GLU versus CON and GLU+DON versus DON). n = 6. (C) The volcano plot (fold change > 2, *P* < 0.05) for DEGs in GLU versus CON and GLU+DON versus DON. Red dots: significantly up-regulated genes, blue dots: significantly down-regulated genes. n = 6. DEG, differential expressed gene; PCA, principal components analysis.


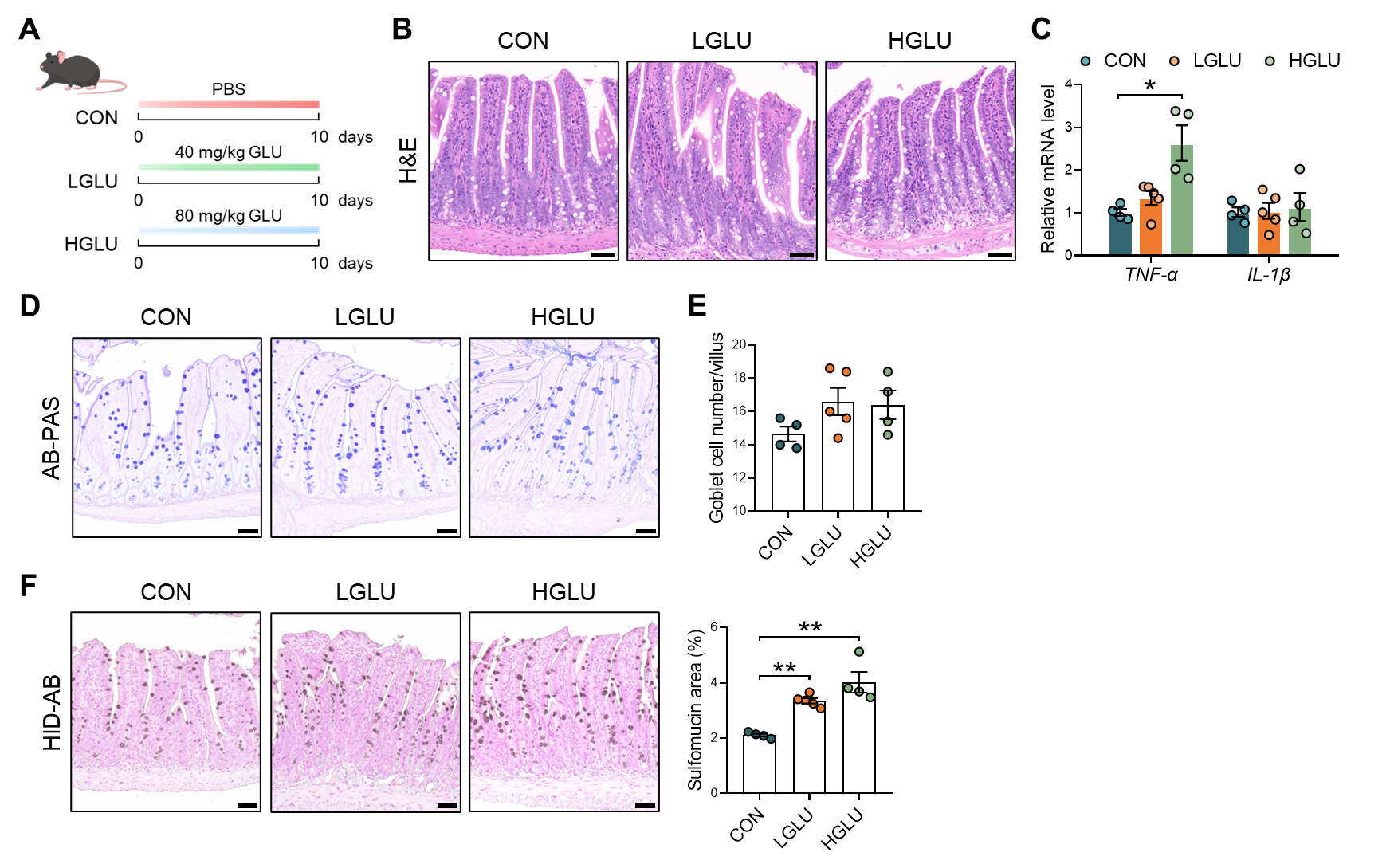


**Figure S2. GLU enhances mucin sulfation in the ileum of mice.** (A) Experimental scheme created with BioGDP.com. (B) Representative images of H&E staining in the ileum. Scale bar: 50 μm. (C) The mRNA expression of inflammatory factor *TNF-α* and *IL-1β* in the ileum. n = 4-5. (D, E) Representative images of AB/PAS staining and the quantification of goblet cells in ileal villus. Scale bar: 50 μm. n = 4-5. (F) Representative images of HID/AB staining and the quantification of sulfomucin area. Scale bar: 50 μm. n = 4-5. Data are presented as mean ± SEM. Statistical analysis was performed using one-way ANOVA with Tukey's multiple comparisons test. ^*^*P* < 0.05 and ^**^*P* < 0.01. H&E, hematoxylin and eosin; TNF, tumor necrosis factor; IL, interleukin; AB/PAS, alcian blue/periodic acid Schiff; HID/AB, iron diamine/alcian blue.


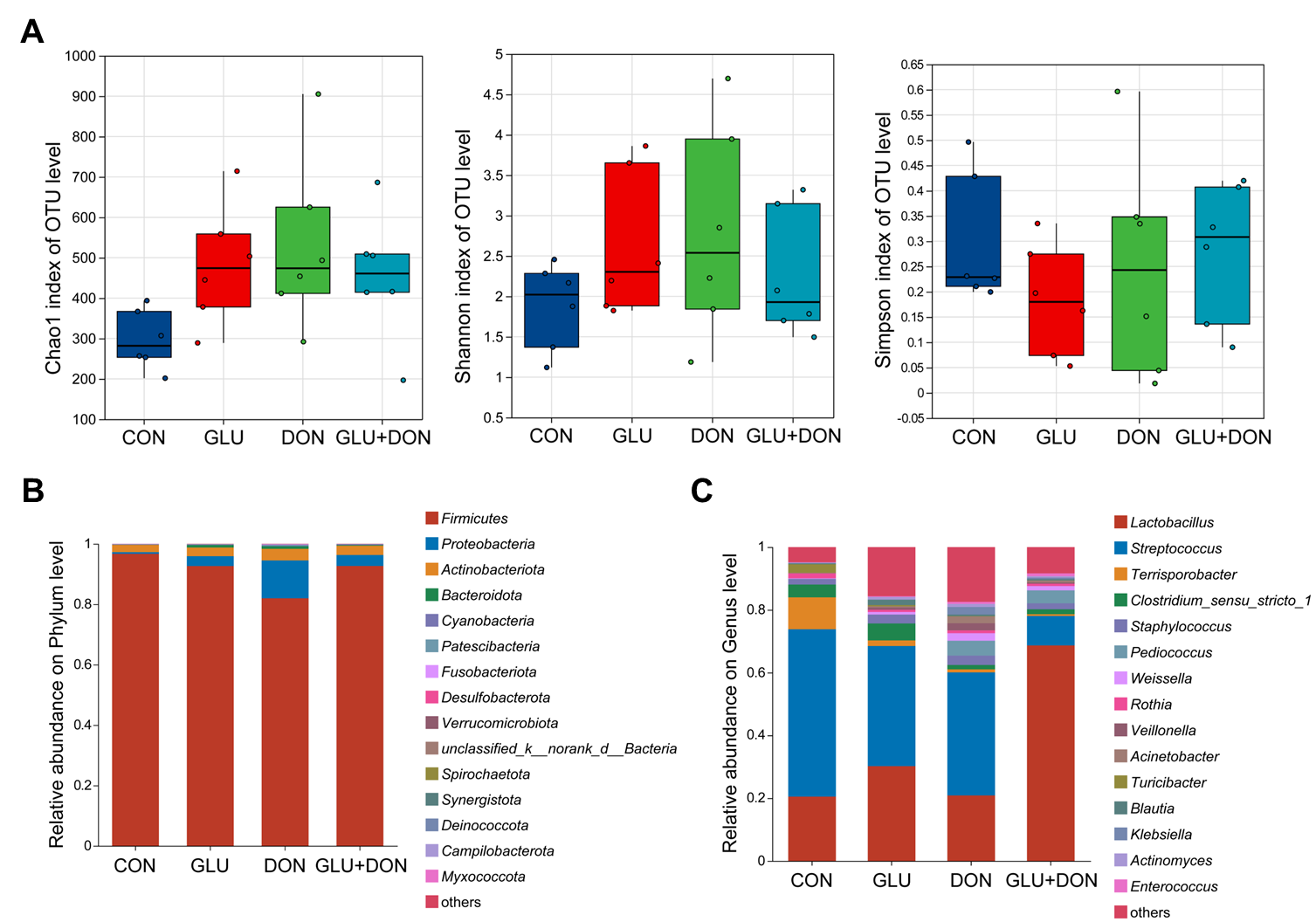


**Figure S3. Microbiome analysis of ileal microbiota in piglets.** (A) α-diversity (assessed by Chao1 index, Shannon index, and Simpson index) of the ileal microbiota in piglets. n = 6. (B, C) The relative bacterial abundance at both phylum and genus levels in ileal microbiota of piglets. n = 6.


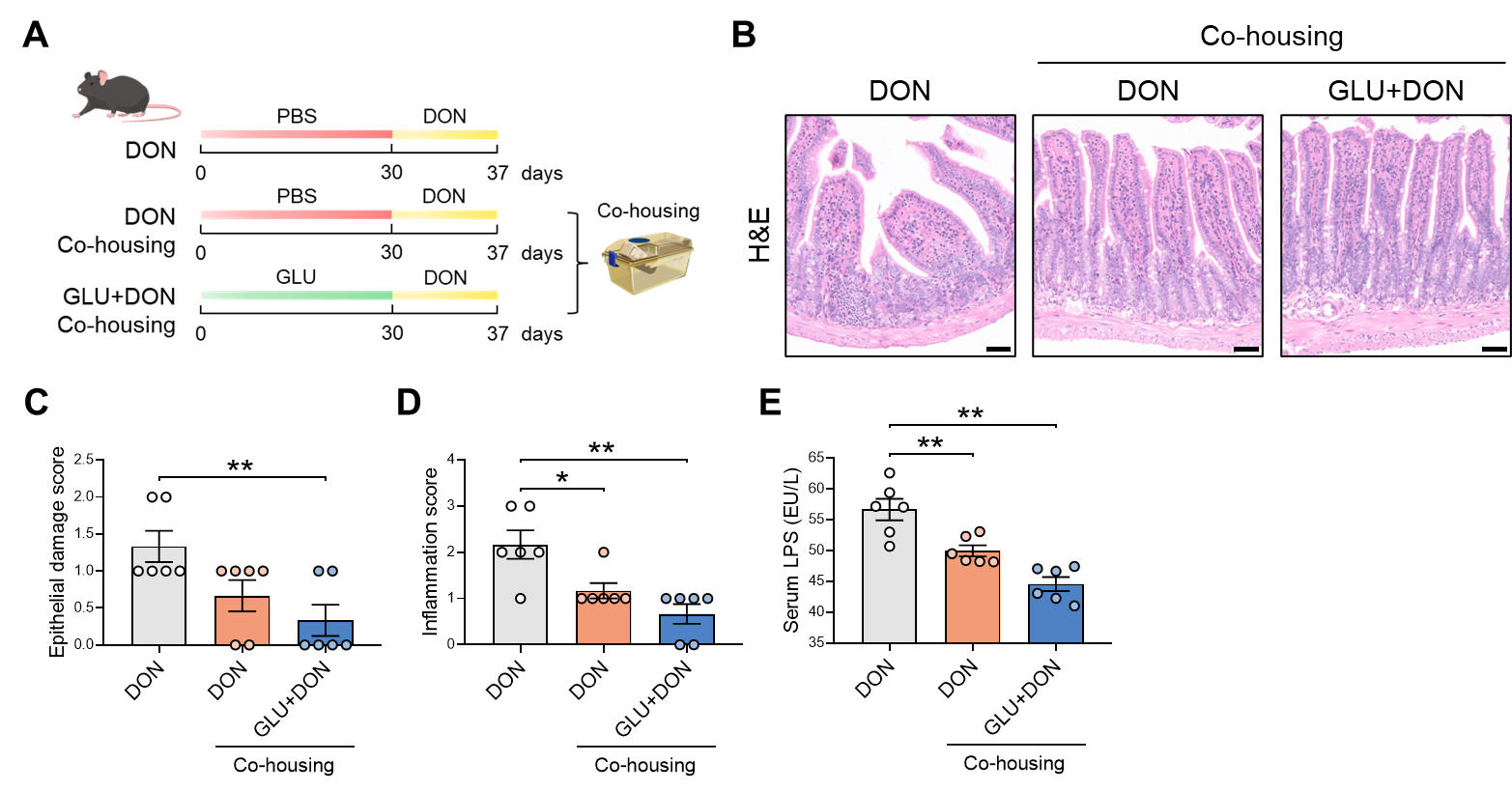


**Figure S4. Co-housing with GLU-treated mice attenuates DON-induced intestinal injury.** (A) Experimental scheme created with BioGDP.com. (B) Representative images of H&E staining in the ileum. Scale bar: 50 μm. (C, D) The epithelial damage score and inflammation score of murine ileum. n = 6. (E) LPS level in murine serum. n = 6. Data are presented as mean ± SEM. Statistical analysis was performed using one-way ANOVA with Tukey's multiple comparisons test. ^*^*P* < 0.05 and ^**^*P* < 0.01. H&E, hematoxylin and eosin; LPS, lipopolysaccharide.


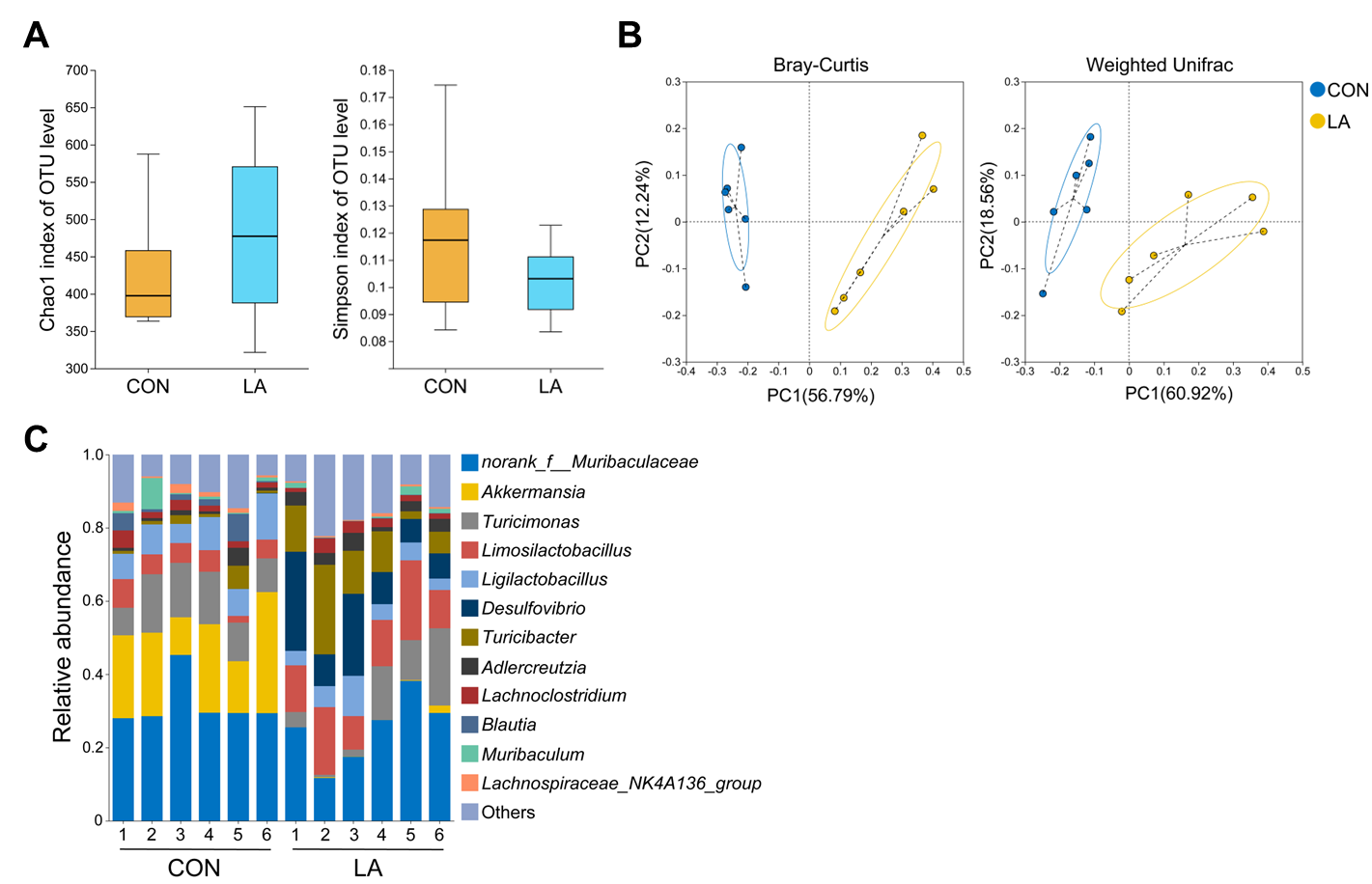


**Figure S5. Analysis of ileal microbiota in mice treated with *Lactobacillus amylovorus*.** (A) α-diversity (assessed by Chao1 index and Simpson index) of the ileal microbiota in the *Lactobacillus amylovorus* experiment. n = 6. (B) The PCoA plot of 16s rRNA sequencing data from murine ileal contents based on Bray-Curtis and weighted Unifrac analysis in the *Lactobacillus amylovorus* experiment. n = 6. (C) The relative bacterial abundance at genus level in the murine ileal microbiota. n = 6. PCoA, Principal coordinate analysis.


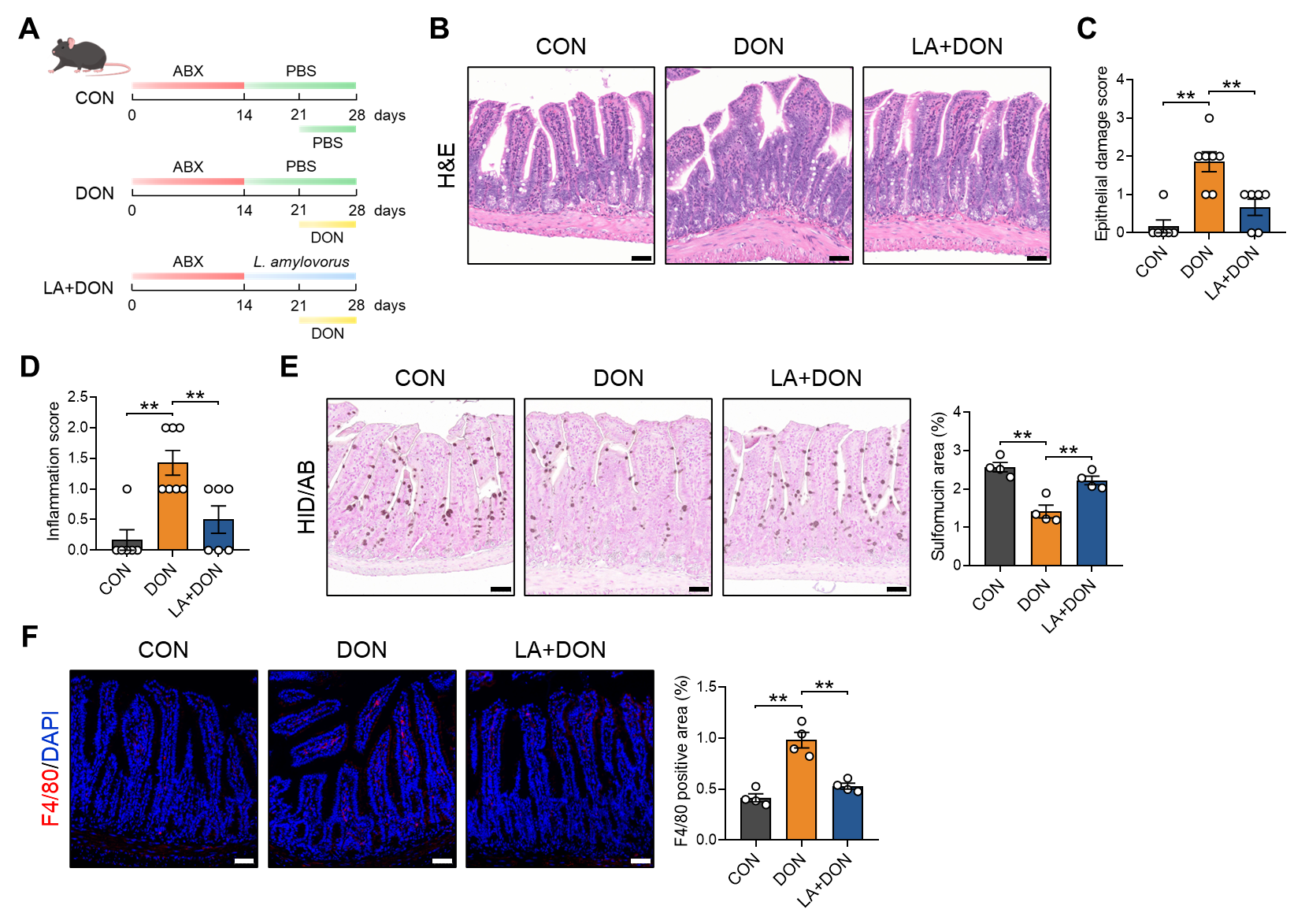


**Figure S6. *Lactobacillus amylovorus* enhances mucin sulfation to alleviate DON-induced intestinal injury.** (A) *Lactobacillus amylovorus* and DON experimental scheme created with BioGDP.com. (B) Representative images of H&E staining in the murine ileum. Scale bar: 50 μm. (C, D) The epithelial damage score and inflammation score in the murine ileum. n = 6-7. (E) Representative images of HID/AB staining and the quantification of sulfomucin area in the *Lactobacillus amylovorus* and DON experiment. Scale bar: 50 μm. n = 4. (F) Representative images of F4/80 staining and the quantification of F4/80 positive area in the murine ileum. Scale bar: 50 μm. n = 4. Data are presented as mean ± SEM. Statistical analysis was performed using one-way ANOVA with Tukey's multiple comparisons test. ^*^*P* < 0.05 and ^**^*P* < 0.01. LA, *Lactobacillus amylovorus*; H&E, hematoxylin and eosin; HID/AB, iron diamine/alcian blue.


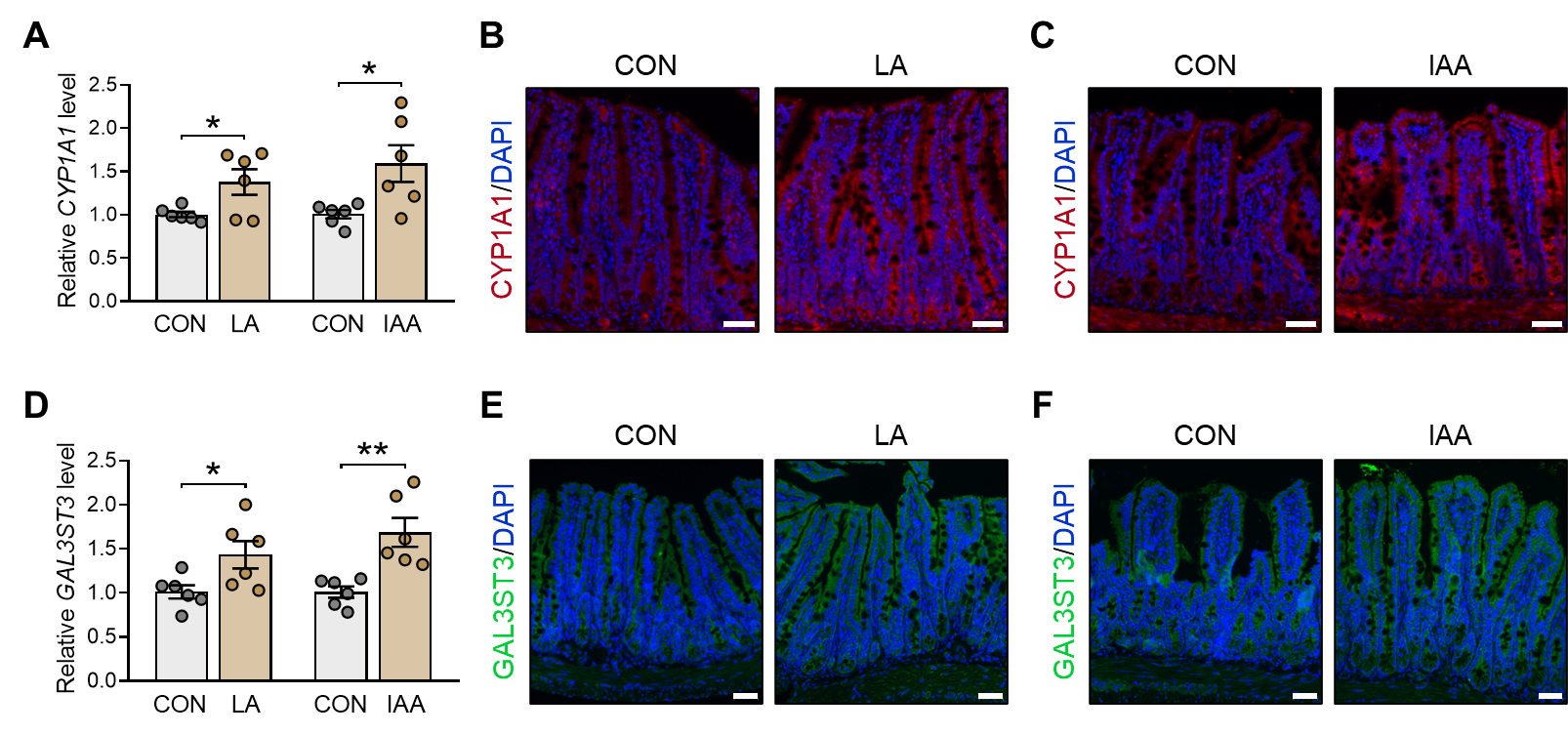


**Figure S7. *Lactobacillus amylovorus* and IAA up-regulate CYP1A1 and GAL3ST3 expression in the ileum.** (A) The CYP1A1 expression in murine ileum in the *Lactobacillus amylovorus* and IAA experiments was examined by qPCR and immunofluorescence. Scale bar: 50 μm. n = 6. (B) The GAL3ST3 expression in murine ileum in the *Lactobacillus amylovorus* and IAA experiments was examined by qPCR and immunofluorescence. Scale bar: 50 μm. n = 6. Data are presented as mean ± SEM. Statistical analysis was performed using unpaired Student’s t-test. ^*^*P* < 0.05 and ^**^*P* < 0.01. LA, *Lactobacillus amylovorus*; IAA, indole-3-acetic acid.


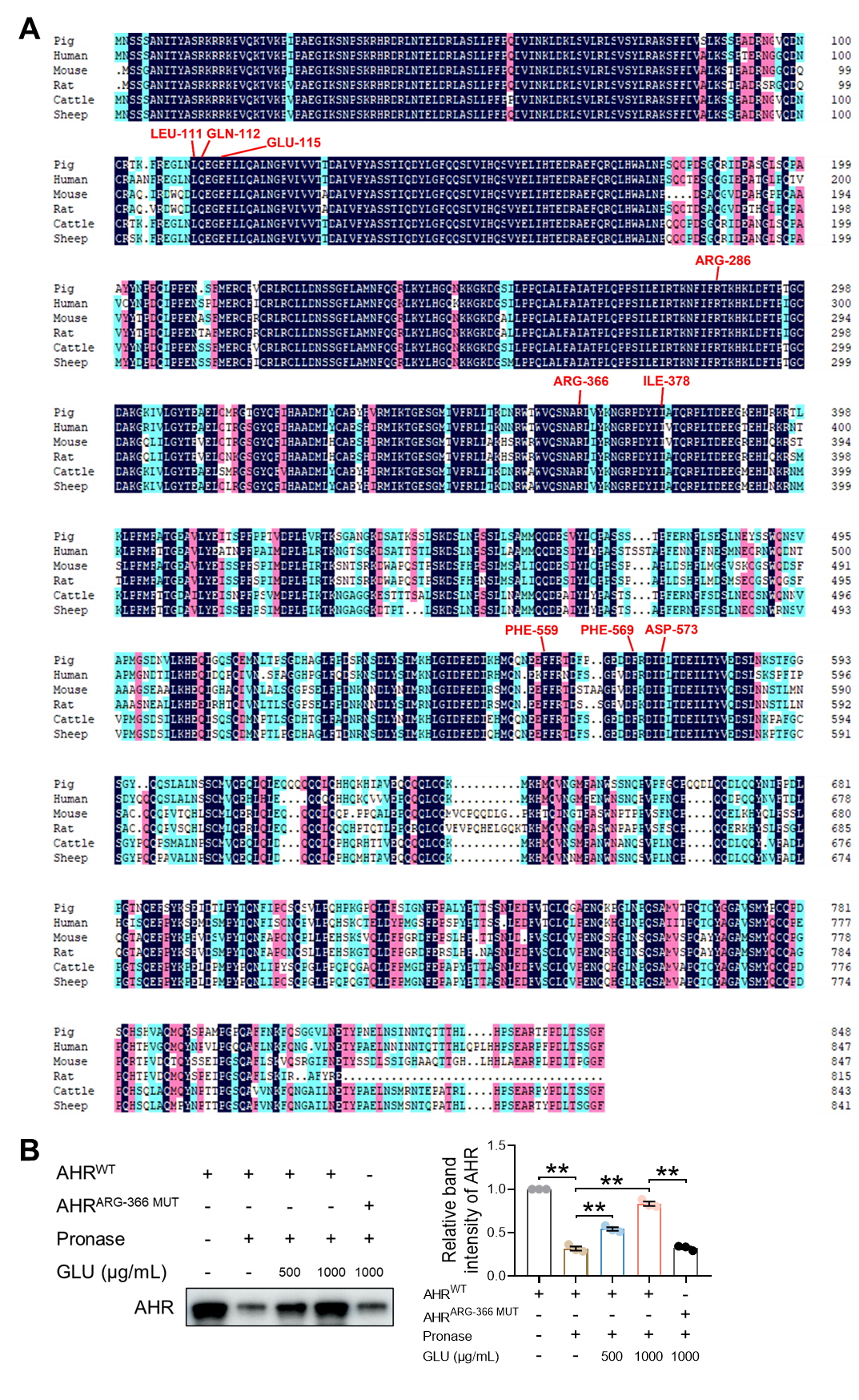


**Figure S8. Sequence homology verification of porcine AHR protein and the binding between GLU and AHR.** (A) Sequence homology verification of AHR protein among pig, human, mouse, rat, cattle, and sheep. (B) DARTS assay was conducted to examine the binding between GLU and AHR. n = 3. Data are presented as mean ± SEM. Statistical analysis was performed using unpaired Student’s t-test. ^*^*P* < 0.05 and ^**^*P* < 0.01. DARTS, drug affinity responsive target stability.


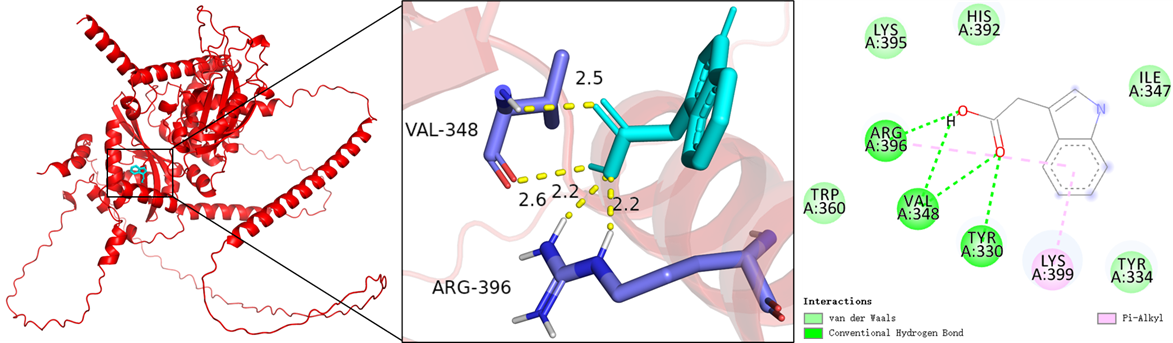


**Figure S9. Molecular docking analysis of IAA and porcine AHR protein.**


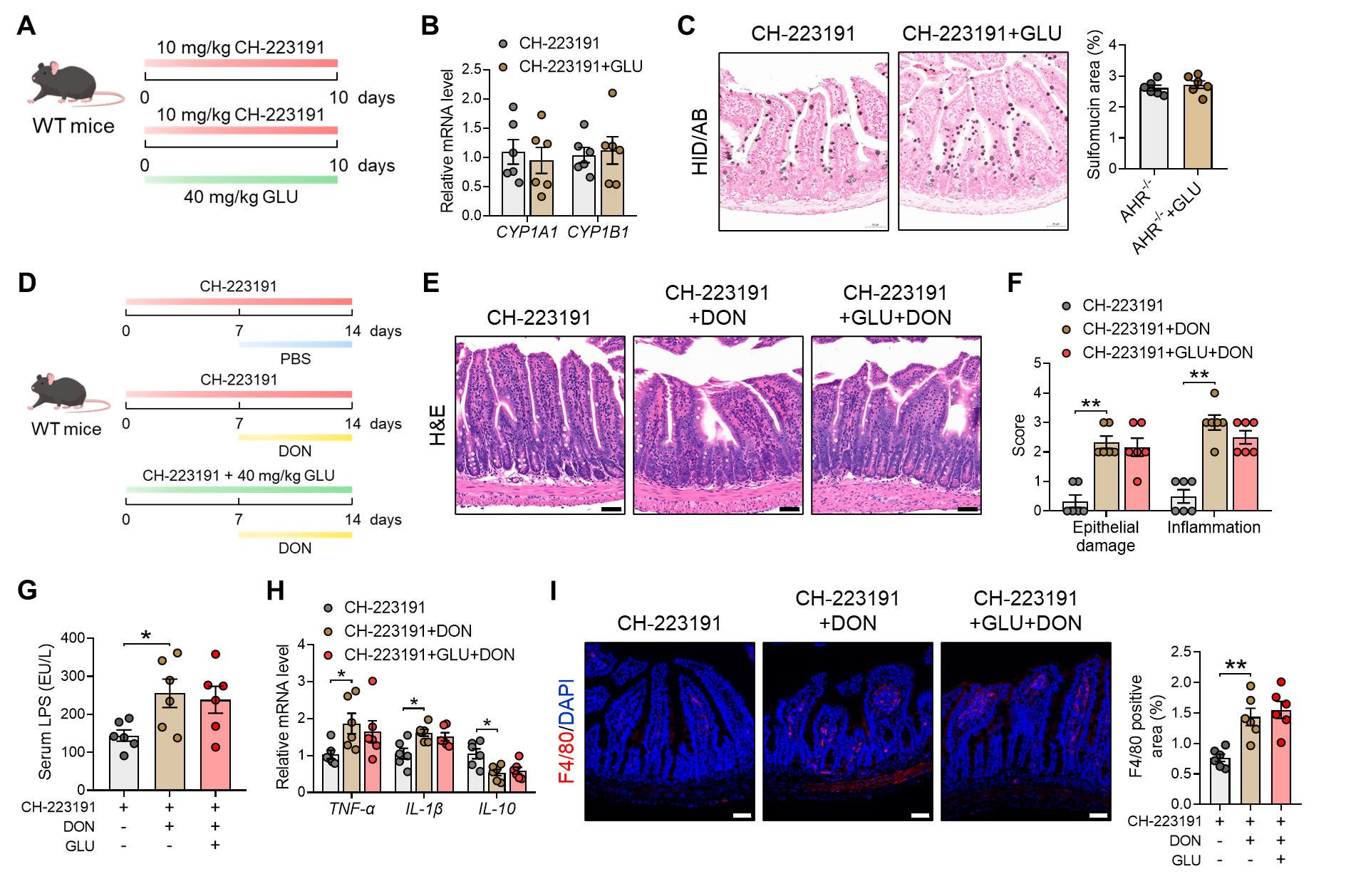


**Figure S10. AHR inhibition by CH-223191 abolishes the protective effects of GLU on mucin sulfation and DON-induced intestinal injury.** (A) CH-223191 and GLU experimental scheme created with BioGDP.com. (B) The mRNA levels of *CYP1A1* and *CYP1B1* in the murine ileum. n = 6. (C) Representative images of HID/AB staining and the quantification of sulfomucin area in the murine ileum. Scale bar: 50 μm. n = 6. (D) CH-223191, GLU, and DON experimental scheme created with BioGDP.com. (E) Representative images of H&E staining in the murine ileum. Scale bar: 50 μm. (F) The epithelial damage score and inflammation score in the murine ileum. n = 6-7. (G) The serum LPS level in mice. n = 6. (H) The mRNA levels of *TNF-α*, *IL-1β*, and *IL-10* in murine ileum. n = 6. (I) Representative images of F4/80 staining (red) and the quantification of F4/80 positive area in the murine ileum. Scale bar: 50 μm. n = 6. Data are presented as mean ± SEM. Statistical analysis was performed using unpaired Student’s t-test and one-way ANOVA with Tukey's multiple comparisons test. ^*^*P* < 0.05 and ^**^*P* < 0.01. HID/AB, iron diamine/alcian blue; H&E, hematoxylin and eosin; LPS, lipopolysaccharide; TNF, tumor necrosis factor; IL, interleukin; DAPI, 4',6-diamidino-2-phenylindole.


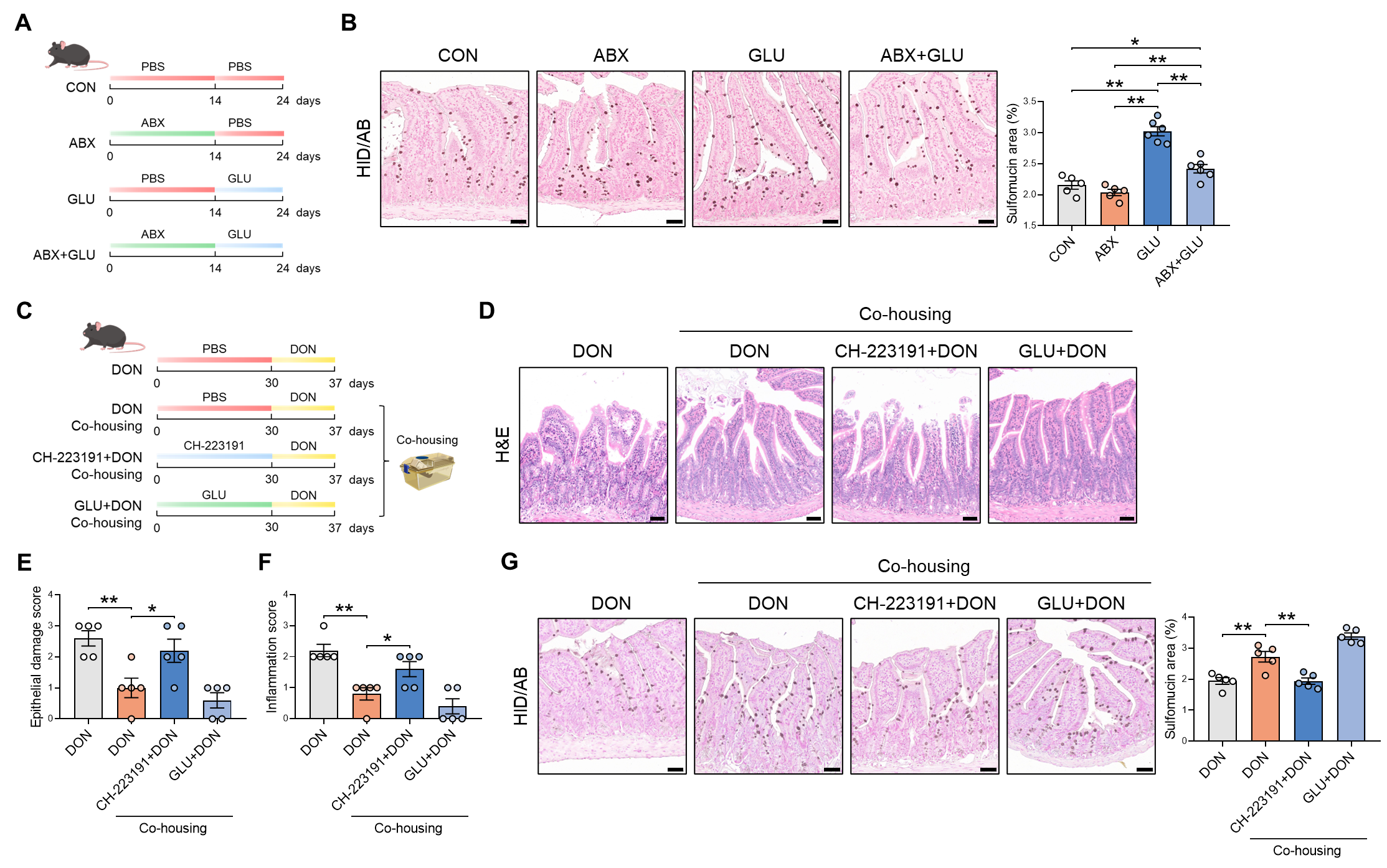


**Figure S11. Blocking IAA signaling weakens the promoting effect of GLU or GLU-associated microbiota on mucin sulfation.** (A) ABX and GLU experimental scheme created with BioGDP.com. (B) Representative images of HID/AB staining and the quantification of sulfomucin area in the murine ileum. Scale bar: 50 μm. n = 5-6. (C) co-housing experimental scheme created with BioGDP.com. (D) Representative images of H&E staining in the murine ileum. Scale bar: 50 μm. (E, F) The epithelial damage score and inflammation score in the murine ileum. n = 5. (G) Representative images of HID/AB staining and the quantification of sulfomucin area in the murine ileum. Scale bar: 50 μm. n = 5. Data are presented as mean ± SEM. Statistical analysis was performed using one-way ANOVA with Tukey's multiple comparisons test. ^*^*P* < 0.05 and ^**^*P* < 0.01. HID/AB, iron diamine/alcian blue; H&E, hematoxylin and eosin.


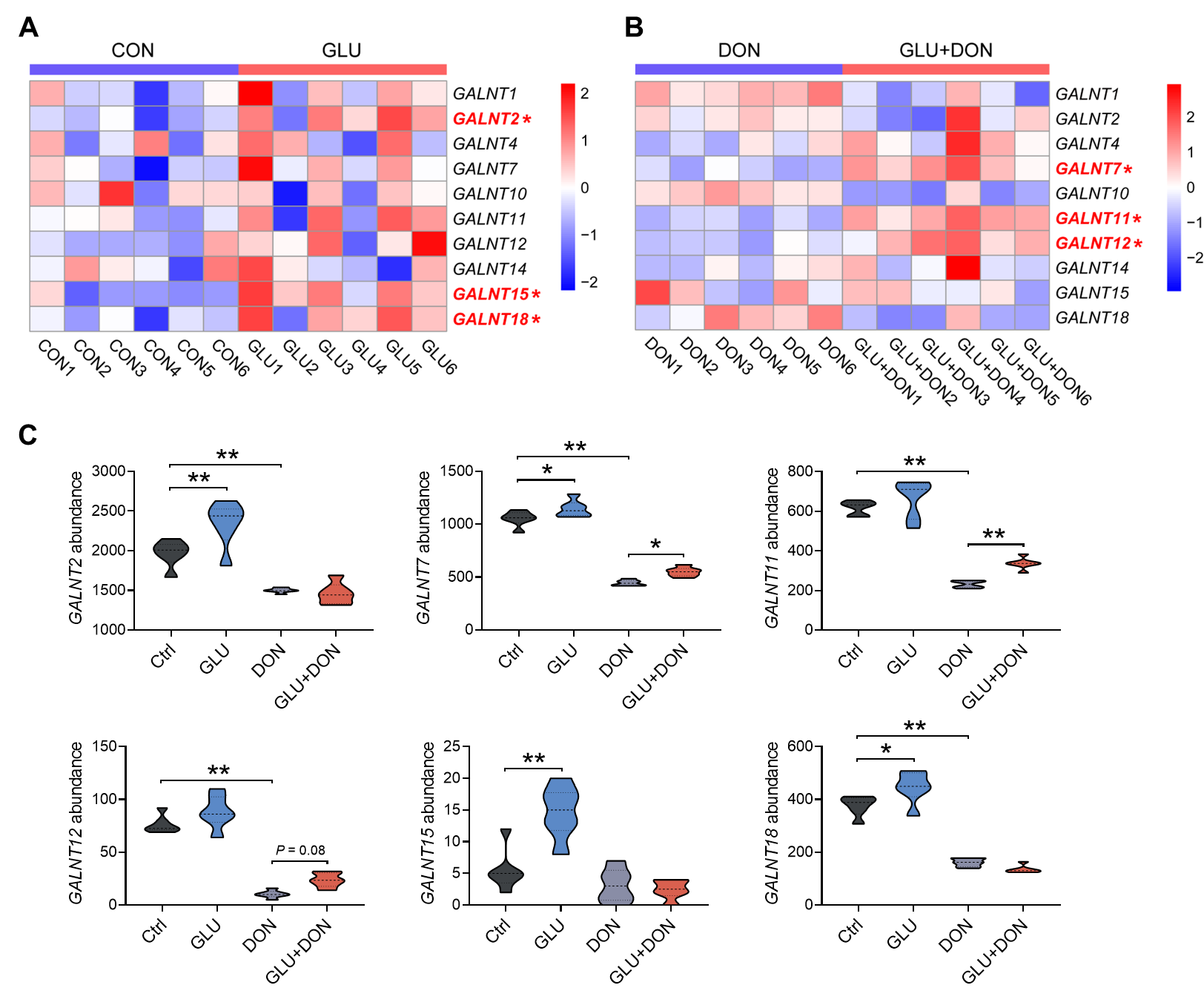


**Figure S12. GLU facilitates the expression of GALNT family protein to promote mucin biosynthesis.** (A) Heatmap of GLANT family genes in porcine intestinal epithelium in GLU versus CON and GLU+DON versus DON. n = 6. (B) The abundances of *GALNT2*, *GALNT7*, *GALNT11*, *GALNT12*, *GALNT15*, and *GALNT18* in porcine intestinal epithelium. n = 6. Data are presented as mean ± SEM. Statistical analysis was performed using one-way ANOVA with Tukey's multiple comparisons test. ^*^*P* < 0.05 and ^**^*P* < 0.01.


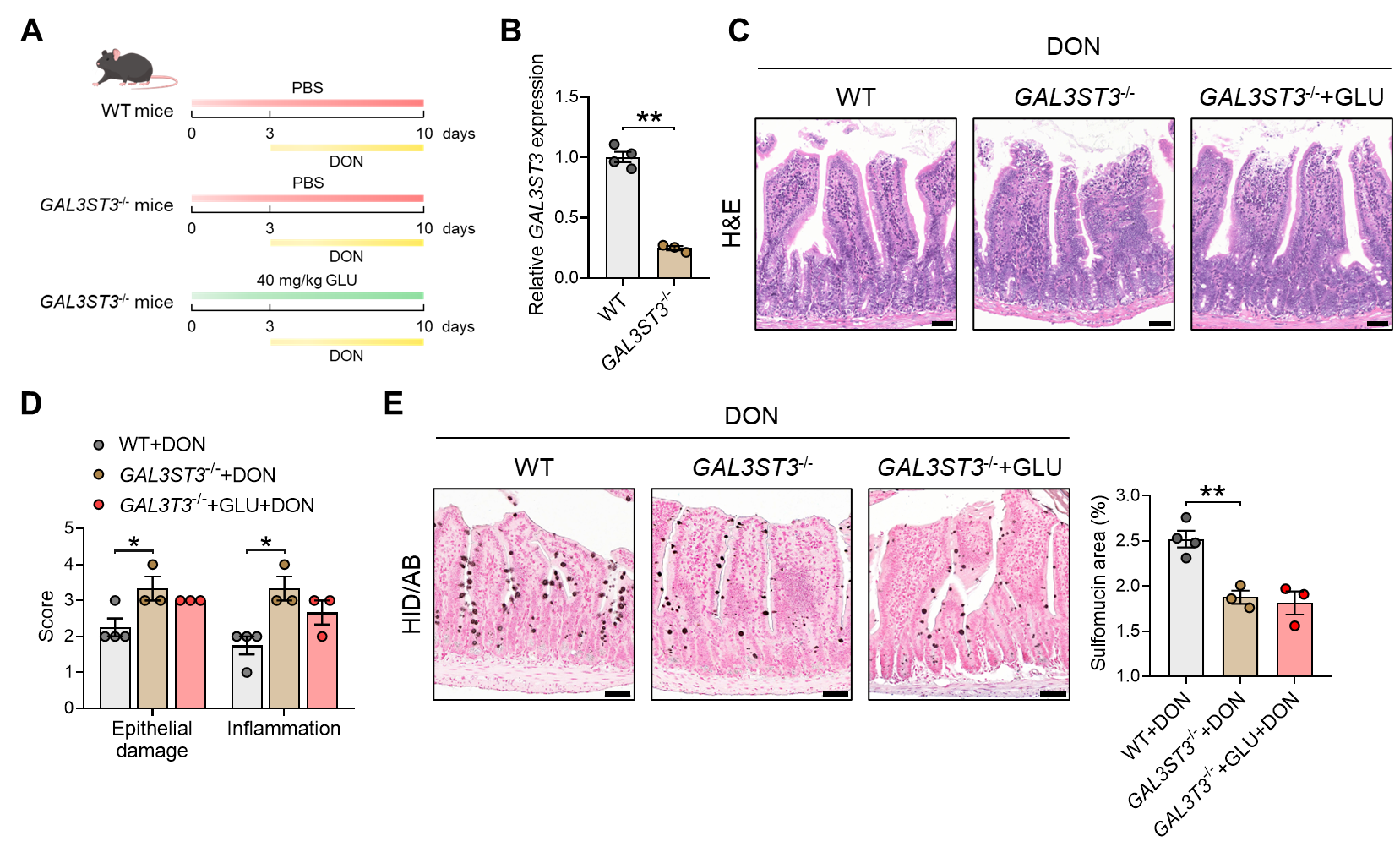


**Figure S13. *GAL3ST3* deficiency abolishes the protective effects of GLU on mucin sulfation and DON-induced intestinal injury.** (A) Experimental scheme created with BioGDP.com. (B) GAL3ST3 mRNA expression in WT and *GAL3ST3^-/-^* mice. n = 3-4. (C) Representative images of H&E staining in the murine ileum. Scale bar: 50 μm. (D) The epithelial damage score and inflammation score in the murine ileum. n = 3-4. (E) Representative images of HID/AB staining and the quantification of sulfomucin area in the murine ileum. Scale bar: 50 μm. n = 3-4. Data are presented as mean ± SEM. Statistical analysis was performed using unpaired Student’s t-test and one-way ANOVA with Tukey's multiple comparisons test. ^*^*P* < 0.05 and ^**^*P* < 0.01. HID/AB, iron diamine/alcian blue; H&E, hematoxylin and eosin.

**Figure S14. GLU metabolite glucuronic acid has no effect on *CYP1A1* and *GAL3ST3* expression.** IPEC-J2 cells were treated with 500 or 1000 μg/mL glucuronic acid for 24 h. Then, the mRNA expression of *CYP1A1* and *GAL3ST3* was examined by qPCR assay. n = 4. Data are presented as mean ± SEM. Statistical analysis was performed using one-way ANOVA with Tukey's multiple comparisons test.


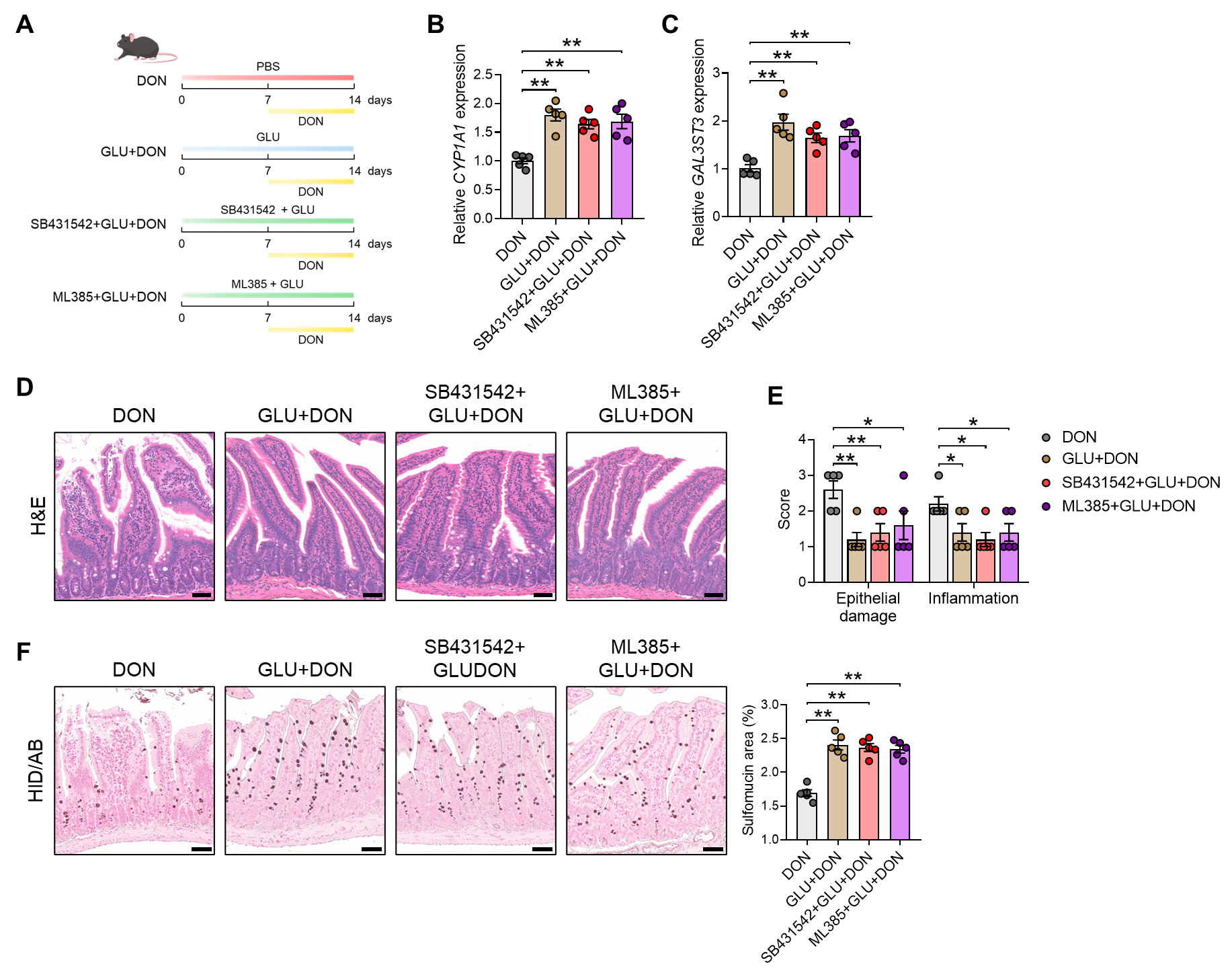


**Figure S15. GLU facilitates AHR/GAL3ST3-mediated mucin sulfation and intestinal protection independently of TGF-β and Nrf2 signaling.** (A) Experimental scheme created with BioGDP.com. (B, C) The mRNA expression of CYP1A1 and GAL3ST3 in murine ileum. n = 5. (D) Representative images of H&E staining in the murine ileum. Scale bar: 50 μm. (E) The epithelial damage score and inflammation score in murine ileum. n = 5. (F) Representative images of HID/AB staining and the quantification of sulfomucin area in murine ileum. Scale bar: 50 μm. n = 5. Data are presented as mean ± SEM. ^*^*P* < 0.05 and ^**^*P* < 0.01. HID/AB, iron diamine/alcian blue; H&E, hematoxylin and eosin.
